# Elbow dimensions in quadrupedal mammals driven by lubrication regime

**DOI:** 10.1038/s41598-023-50619-x

**Published:** 2024-01-25

**Authors:** Kalenia Marquez-Florez, Santiago Arroyave-Tobon, Loïc Tadrist, Jean-Marc Linares

**Affiliations:** Aix Marseille Univ, CNRS, ISM, Marseille, France

**Keywords:** Biomedical engineering, Mechanical engineering, Statistical methods, Skeleton

## Abstract

Synovial joints, such as the elbow, experience different lubrication regimes, ranging from fluid film to boundary lubrication, depending on locomotion conditions. We explore the relationship between the elbow lubrication regime and the size of quadrupedal mammals. We use allometry to analyze the dimensions, contact stress, and sliding speed of the elbow in 110 quadrupedal mammals. Our results reveal that the average diameter and width of the distal humerus are scaled $\propto M^{0.35}$, which allowed us to estimate a consistent contact pressure and sliding speed across mammals. This consistency likely promotes fluid film lubrication regardless of body mass. Further, the ratio between the diameter and width is about 0.5 for all analyzed taxa, which is a good compromise between loading capacity and size. Our study deepens our understanding of synovial joints and their adaptations, with implications for the development of treatments, prostheses, and bioinspired joint designs.

## Introduction

Synovial joints allow for relative movement of connected bones and fulfill weight-bearing functions. They collectively contribute to animal movement while minimizing energy loss due to friction^1^. This low friction is attributed to the lubrication system of the joint, which limits tissue damage. Any breakdown of the components involved in the joint lubrication is associated with joint disorders and pathologies^2,3^.

Understanding lubrication in synovial joints requires an extension of the principles of tribology, an engineering discipline that examines interactions between surfaces in relative motion. In general terms, lubrication conditions fall under various regimes, ranging from boundary to fluid film lubrication. In the fluid film regime, the conditions allow for a lubricant film between the interacting surfaces, preventing contact between asperities. This mechanism prevents friction and wear, in contrast to the boundary regime^4,5^. The lubrication regime experienced by a joint is influenced by various variables: fluid and material variables (synovial fluid viscosity and cartilage material properties, which are consistent across taxa^6–8^), and operational variables (applied normal load distributed in the joint, and the relative speed between the interacting surfaces, i.e., average contact stress and sliding speed). These operational variables, in turn, are conditioned by geometrical variables (dimensions of the interacting surfaces representing the joint), which are linked to animal size.

Over decades, researchers have extensively investigated synovial joint lubrication from both theoretical^1,4,9–18^, and experimental^2,3,19,20^ perspectives, focusing in humans and specific animals species. However, the existing literature still presents challenges in inferring the relationship between geometrical and operational variables regarding lubrication behavior across taxa. For instance, are the lubrication conditions in a small mouse comparable to those in a large elephant? Understanding these relationships may pave the way for the development of innovative bioinspired mechanical joints and treatments for joint-related pathologies. The optimal synovial joint function should prioritize the fluid film lubrication regime, as it is known to reduce cartilage wear and tissue breakdown^1^. If so, we hypothesize that joint dimensions adapted to the locomotion operational conditions favor the fluid-film lubrication regime. As fluid and material variables are both consistent across taxa, our hypothesis implies that joint dimensions should allow the sliding speed and average contact stress to also be consistent across taxa.

**Figure 1:**
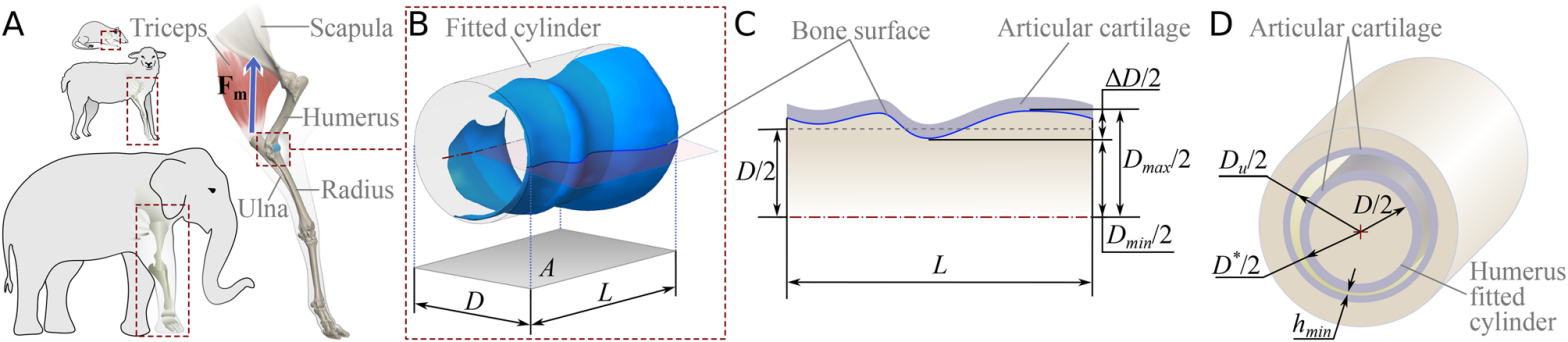
Analyzed dimensions on the distal humerus. (**A**) Forelimb of a *Ovis orientalis aries*. The triceps is represented with its resultant force, $\mathbf {F_m}$. (**B**) Distal articular surface of the *Antilope cervicapra* humerus (object: MNHN:ZM:AC-1901-174, media ID: 000397840 from MorphoSource.org) with its fitted cylinder. The gray-colored region (with area *A*) is a projection of the fitted cylinder, along with the distal humerus average diameter, *D*, and width, *L*. (**C**) Profile of the distal humerus with the minimum diameter, $D_{min}$, maximum diameter, $D_{max}$, diameter difference ($\Delta D/2$), and the average diameter *D*. (**D**) Elbow equivalent cylindrical surfaces in conformal contact, where $D^{*}$ is the humerus diameter including the cartilage thickness, $D_u$ the diameter of the opposing surface, and $h_{min}$ the minimum synovial fluid film thickness during elbow extension. $D_u$ and the cartilage thickness were assumed to be proportional to *D*, based on values reported for the ankle^12^.

Here, we evaluate this hypothesis for the distal humerus in a sample of 110 quadrupedal mammals. This bone is crucial for locomotion^21,22^, limb posture^23,24^, and linked to ecological adaptations^25–30^. Utilizing the dimensions of the distal humerus and the size of the animals, we estimate operational variables (i.e. sliding speed and average contact stress) during galloping. Among the different gaits of quadrupedal mammals, galloping induces the highest extension speeds of the elbow^31–33^. We then extend the analysis to assess the lubrication regime, considering the estimated lubricant film thickness, for a simplified geometry of the elbow joint (modeled as cylinders in conformal contact, each with a layer of cartilage and an associated rugosity; see Fig. 1). Our analysis suggests that elbow dimensions consistently promote fluid film lubrication by maintaining consistent average contact stress and sliding speed across quadrupedal mammals. Our study deepens our understanding of synovial joints and how they endure functional requirements.

## Results

### Allometry of the distal humerus dimensions

**Figure 2:**
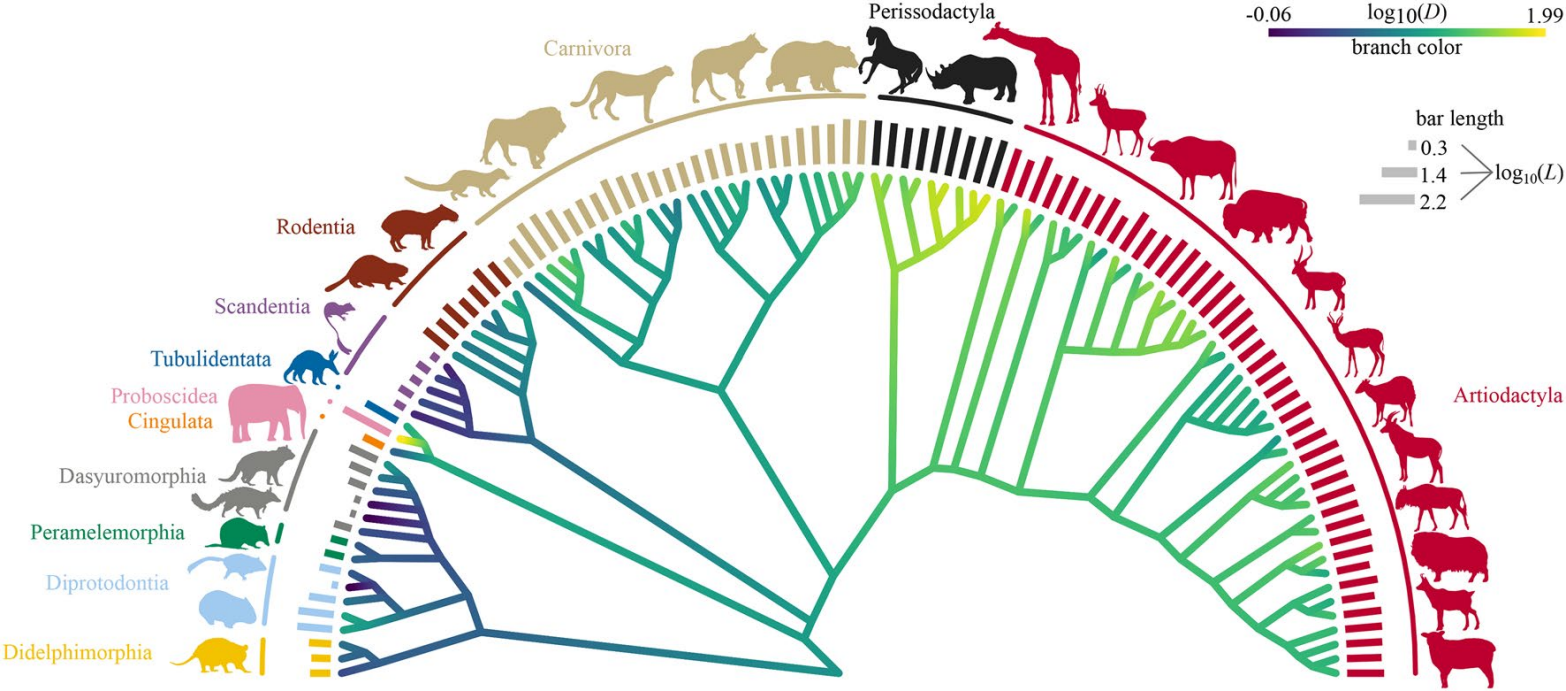
Phylogenetic tree with an overviewing of the taxa examined in the study. Branches are color-coded based on the $\log _{10}$-transformed average diameter $\log _{10}(D)$, but values other than those at the tip of the branches are approximate and do not support any conclusions. The outer bars length indicates the $\log _{10}$-transformed width $\log _{10}(L)$. The image was produced using modified branch lengths to improve visualization (with the function compute.brlen from the ’ape’ package for R^34^ following Grafen’s method with a power of 0.65). The tree was plotted with the ’phytools’ package for R^35^. Silhouettes from PhyloPic (see Supplementary Information (SI) for silhouette credits).

The analyzed animals ranged from 0.02 kg to 4000 kg, (see Fig. 2 and SI-Table S2). The measured average diameter, *D*, of the distal humerus, ranged from 0.87 mm to 98.55 mm, and the width of the distal humerus, *L*, ranged from 2.1 mm to 164.9 mm (Table S1). Figure 1 shows the dimensions considered in this study.

**Figure 3:**
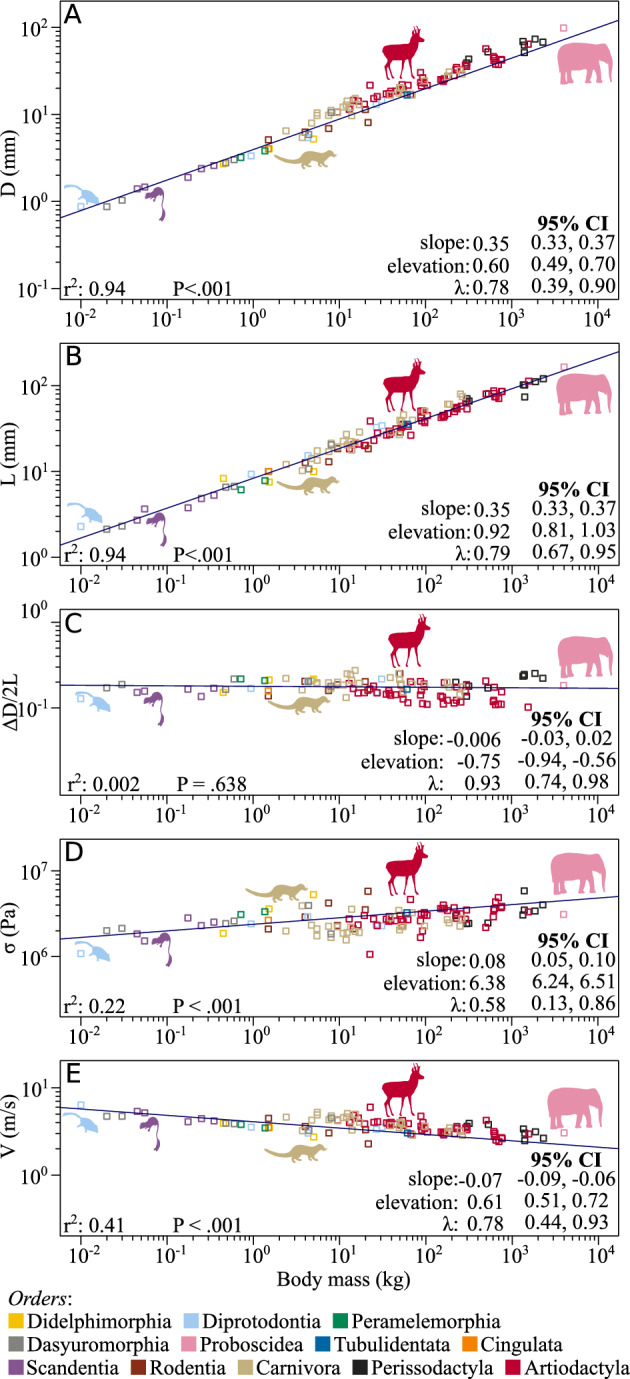
Allometry of analyzed parameters. (**A**) average diameter, *D*; (**B**) width, *L*; (**C**) ratio $\Delta D/2L$; (**D**) average contact stress, $\sigma$; (**E**) sliding speed *V*. Slopes and elevations were estimated from phylogenetic generalized least squares (PGLS) regressions. The species (points) are color-coded according to their order. Silhouettes are from PhyloPic: *Acrobates pygmaeus* by Sarah Werning; CC BY 3.0; *Ptilocercus lowii*, Public Domain Dedication (PDD); *Genetta genetta*, Public Domain Mark 1.0; *Antilocapra americana*, PDD; *Elephas maximus*, PDD.

We used phylogenetic generalized least squares (PGLS) analyzes to find the correlations of *D* and *L* with body mass, *M*: $D \propto M^{0.35}$ and $L \propto M^{0.35}$ (Fig. 3A and B). Interestingly, the ratio between *D* and *L* was found to be independent of the body mass, with $D/L \approx 0.5$ (confidence interval (ci):(0.4, 0.8)). The results indicated a strong correlation between these variables, where body mass explains 94% ($P<.001$) of the variations with a strong influence of the phylogenetic history (*D*: $\lambda =0.78$; *L*: $\lambda =0.79$; see Table S4).

We evaluated the ratio of the change in the radius, $\Delta D/2$ (Fig. 1), to the width of the distal humerus joint, *L*, to estimate the aspect ratio of the morphology of the articular surface profile: $\Delta D / 2L$. The PGLS analysis showed no correlation between this ratio and body mass with a strong phylogenetic signal ($r^2 = 0.002$, $P =.638$, $\lambda = 0.93$), and gives a small allometric slope of $-0.01$, suggesting independence of this ratio from body mass (Fig. 3C).

### Estimated averaged contact stress $\sigma$

We defined $\sigma$ as $F_{j}/ A$, which is interpreted as the force at the joint, $F_{j}$, distributed uniformly on the area of the projection of the fitted cylinder: $A = L D$ (Fig. 1). We approximated $F_{j}$ to the triceps concentric maximum force, $F_m$ (see *asm4.*). $F_m$, was estimated as $A_m \sigma _m$, where $A_m$ is the muscle cross-sectional area and $\sigma _m$ the maximum stress the muscle can generate – consistent among vertebrates (about $0.2-0.3$ MPa, here $\sigma _m = 0.25$ MPa)^36^. $A_m$ was estimated using the muscle mass, $m_m$, fiber length, $L_m$, and density, $\rho _m$: $A_m = m_m /(L_m \rho _m)$^36^. In mammals, the triceps mass and fiber length allometries are $m_m = 6.2M^{1.11}$ (for the muscle mass in g and the body mass in kg) and $L_m = 18.7M^{0.33}$ (for fiber length in mm and the body mass in kg)^37^. $\rho _m$ is consistent among mammals at about $1060\hbox {kg/}\hbox {m}^{3}$^37^. From above, $F_m = 78.20M^{0.78}$ (for $F_m$ in N and *M* in kg). Further, $\sigma \propto M^{0.08}$ (Fig. 3D), thus, $\sigma$ is consistent across all taxa, at about $2.4 \pm 0.75$ MPa. This is confirmed by the small correlation coefficient revealed by the PGLS regression ($r^2 = 0.23$, $P < .001$, $\lambda = 0.58$). For detailed statistics see Table S4.

### Estimated maximum sliding speed *V*

We defined *V* as the relative speed at the joint average radius, *D*/2, during the rapid extension of the elbow; $V = \omega ~ D/2$. The angular speed, $\omega$, depends on the angular acceleration $\alpha$ and the joint excursion time, *t*, as $\omega = \alpha t$. The triceps force $F_m$ (see *asm4.*) generates the acceleration, $\alpha$, which is inversely proportional to the forearm’s moment of inertia, *I*: $\alpha = F_m~k/I$, where *k* is the moment arm of $F_m$. For the triceps, $k = 8.7~M^{0.41}$ (*k* in mm and the body mass in kg)^37^. The moment of inertia at the elbow of the forearm scales as $I = 1.77 \times 10^{-5}~M^{1.78}$ (*I* in $\hbox {kg m}^{2}$ and *M* in kg, see *asm6.* and SI-Fig. S3). The joint excursion time, *t*, is inversely proportional to the stride frequency, $S_f$: $t \propto S_f^{-1}$. For gallop, $S_f$ is independent of speed and scales allometrically with body mass as $S_f = 4.70M^{-0.16}$ ($S_f$ in Hz and body mass in Kg)^38^. According to *asm5.*, *t* is a percentage of the stride period in galloping (specifically, $25.4\%~ S_f^{-1}$, see SI-Fig. S1). From above, $V \propto M^{-0.07}$ (Fig. 3E), thus, is consistent across all taxa, at about $4.1 \pm 0.1$ m/s. The PGLS revealed a weak correlation between *V* and body mass ($r^2 = 0.41$, $P<.001$, $\lambda = 0.78$). For detailed statistics see Table S4.

### Estimated minimum lubricant film thickness $h_{min}$

In dynamically loaded joints, the lubrication mechanism is isoviscous-elastic, as cartilage deforms easily and the viscosity of synovial fluid varies little under physiological loads^3,5,13,39^. Our modeling approach represents the elbow as two cylinders in conformal contact, each with diameters $D^{*}$ and $D_u$ (Fig. 1D). The modeling considers the presence of a layer of cartilage coating the cylinders, as well as the clearance between them (see Fig. 1D). Since we assume a constant cartilage thickness along the cylinders, $D^{*}$ and $D_u$ are functions of the average diameter, *D*, along with two proportionality parameters, $c_i$ and $c_{ii}$, which define the articular cartilage thickness and the clearance, respectively (see SI-Fig. S7). Consequently, we express them as follows: $D^{*} = D(1+c_i)$ and $D_u = D(1+c_i+c_{ii})$. Based on measurements reported in the literature^12^, we estimate that these values should be around $c_i = 0.06$ and $c_{ii} = 0.07$. However, due to the lack of sufficient information in the literature to estimate the values of $c_i$ and $c_{ii}$, we conducted a sensitivity analysis to assess their impact on the lubricant film thickness (see SI-Fig. S8).

For tow cylinders in conformal contact, the minimum lubricant film thickness that separates the surfaces is given by $h_{min} = R_x(7.43U^{0.65}W^{-0.21}$)^5^. The effective radius, $R_x$, the dimensionless speed parameter *U*, and the dimensionless load parameter *W*, were estimated considering: $E = 8.1$ MPa^6^, $\nu = 0.4$^7^, and $\eta = 0.01$ Pa$\cdot$s^8^. $R_x$ is defined as $R_x = D^{*}D_{u}/(2(D_{u}-D^{*}))$ (see Fig. 1); *U* as $\eta V^{*}/(E'R_x)$, where $V^{*}$ is the sliding speed at $D^{*}$ and $E'$ is the effective Young’s modulus: $E'=E/(1-\nu )$. *W* is defined as $F_m/(E'R_x^2)$. These equations give $h_{min}\propto M^{0.06}$, independent of body mass, estimated at about $24\,\upmu \hbox {m}$.

## Discussion

We used allometry to analyze distal humerus dimensions, allowing us to estimate average contact stress, maximum sliding speed, and lubricant film thickness in 110 quadrupedal mammals. We estimated that the average contact stress and maximum sliding speed were consistent across taxa, aligning with the classical theories of allometry^40^ (i.e. geometric similarity, elastic similarity, and constant stress similarity -see SI-Table S8). If so, this consistency allows for a constant minimum lubricant film thickness across taxa, ensuring a consistent lubrication regime. Therefore, a small mammal such as *Acrobates pygmaeus* (13 g) and a large one such as *Elephas maximus* (4000 kg) may experience similar lubrication conditions at the elbow during rapid extension of the joint.

The fact that joint fluid and material properties are similar across taxa^6–8^ suggests that joint dimensions likely evolved for tissue maintenance. This agrees with existing literature that highlights the adaptability of bone morphology to mechanical stimuli during development^41,42^. Specifically, the consistent ratio $D/L \approx 0.5$ of the distal humerus strikes a balance between loading capacity and joint width. This ratio resembles industrial long bearings ($D/L \le 0.5$), where lubricant film pressure varies little along the rotation axis, maximizing lubricant loading capacity and minimizing lubricant outflow^43^.

The joint operational variables of maximum sliding speed and average contact stress were estimated consistent across taxa. Despite the simplification of the elbow as two cylinders, the sliding speed at a given point of the joint surface is consistent across taxa due to the constant aspect ratio of the joint profile ($\Delta D/2L$). The average contact stress, estimated at 2.4 MPa, falls within the range of intermittent compressive hydrostatic stress for chondrocytes to stay healthy ($1-10$ MPa)^44^. Our findings align with those of Brand^45^, who inferred from literature data of four mammal species that the average contact stress ranged from $0.1-2.9$ MPa regardless of the joint.

Further, the quadrupedal mammal elbow might have adapted to attain fluid film lubrication under extreme locomotion, as in galloping. The estimated maximum sliding speed coupled with the estimated average contact stress promotes a minimum lubricant film thickness at about 24 $\upmu \hbox {m}$ for all analyzed taxa. In engineering, we use the film parameter $\Lambda$ to characterize the lubrication regime. This parameter compares the minimum lubricant film thickness, $h_{min}$, to the combined roughness of the surfaces in contact, $R_a$: $\Lambda = h_{min}/\sqrt{2}R_a$^5^. In the traditional classification: $\Lambda < 1$ is boundary lubrication, $1 \le \Lambda < 3$ is mixed lubrication and $\Lambda \ge 3$ is fluid film lubrication. As $R_a$ for the articular cartilage has been measured at 1.42 $\upmu \hbox {m}$^8^, $\Lambda$ is at about $12\gg 3$. This suggests fluid film lubrication, with some security margin, which might ensure this lubrication during galloping even under conditions of high surface roughness (see SI-Figs. S9–S11). Additionally, local pressures can smooth out surface asperities, allowing fluid film lubrication even if $\Lambda < 1$^14,46^. SI-Figure S9 details this modeling and evaluation of film thickness and lubrication regime while considering variations in the conformal contact hypothesis (i.e., changes in cartilage thickness and clearance between cylinders).

The findings of this study should be interpreted mindful of the assumptions. We estimated contact stress and sliding speed values based on measurements of distal humeri dimensions, coupled with allometric expressions from the literature, which may introduce potential uncertainties. Assumptions regarding the triceps force reaction time^47^ and neglecting antagonist muscles^31,47^ may slightly affect the estimated maximum sliding speed. Hence, in SI-Figure S9 we further evaluated the combined effect of smaller sliding speeds and different roughnesses of the cartilage on the lubrication regime. While fluid film lubrication is maintained for small to moderate velocities and roughness, it shifts to mixed lubrication at very small sliding speeds ($< 0.17$ m/s) and to boundary lubrication for extremely low speeds ($< 0.014$m/s). However, factors such as squeeze-film action and surface properties of the cartilage become significant at low speeds, which influence the lubrication regime^8^. Furthermore, our simplified model treated the elbow as two cylinders in contact, overlooking the complex morphology of the elbow. We also assumed an allometric similarity of cartilage thickness and joint clearance to the average diameter, potentially impacting the estimated lubrication regime. To assess the sensitivity of the regime to variations in cartilage thickness and joint clearance, we conducted a detailed analysis in SI-Figs. S8–S11. Additionally, while studies indicate a subperiosteal transmission of pressure^48^, we opted to neglect this pressure due to its significant difference in magnitude compared to joint contact pressures, however, it might be considered in future works.

In conclusion, our study provides insight into the workings of natural joints. Our findings suggest that the dimensions of the distal humerus might have evolved so that stresses and sliding velocities promote fluid film lubrication, beneficial for tissue maintenance. This sizing strategy is similar to the bushing design in engineering, where dimensions rely on the ability of the material to withstand pressures and velocities. Ultimately, this study might eventually contribute to bioinspired joint designs.

## Methods

### Data collection

We obtained 3D-reconstructed humeri of quadrupedal extant mammals from the MorphoSource.org database. Only bones with ossified growth plates were considered for the analysis. Knuckle-walking mammals and primates were excluded due to differences in locomotion style and forelimb weight support during gait^49^. Our dataset included 203 bones from 110 species across 35 families and 12 mammalian orders (SI-Table S1). We did not differentiate between the gender or lateral side of the bones. We searched published literature to obtain the average mass of the animals in the study (SI-Table S2).

### Assumptions

We considered the following assumptions for the development of this study:

*asm1.*: The distal humerus articular surface can be approximated as a revolution surface, and the elbow was simplified as two cylinders in conformal contact^50,51^.
*asm2.*: The variables are independent of each other if the magnitude of the allometric exponent is less than 0.1^38^.
*asm3.*: We analyzed the conditions of a rapid extension of the elbow which occurs naturally during galloping. In this gait, the stride frequency ($S_f$) only depends on the body mass^38^.
*asm4.*: In galloping (see *asm3.*), the triceps exhibits strong activity during elbow extension, surpassing the biceps and brachialis during flexion^31–33^. The triceps uses elastic energy storage during locomotion to reduce the work done at the shoulder^24,33,52^. Further, during galloping, muscles perform their maximal force which determines the ground reaction force and the loads on bones and joints^53^. In that line, we assumed that: i) the triceps is in charge of the rapid extension of the elbow; ii) the force at the joint is similar to that of the triceps.
*asm5.*: The duration of the extension periods was determined as a percentage of the stride using data from Tokuriki^31^ and the PlotDigitizer tool. On average, the extension instances make up $25.4\%$ of the stride period (see SI-Fig. S1).
*asm6.*: Using supplementary data from Coatham et al.^54^(who conducted virtual segmentation of computed tomography scans of animal skins), we calculated the moment of inertia, *I*, at the elbow of the lower arm (arm and hand) of quadrupedal mammals. We obtained $I = 1.77 \times 10^{-5}~M^{1.78}$ (for *I* in $\hbox {kg m}^{2}$ and the mass in kg)(see SI-Fig S3).

### Specimen measurements

We used the software 3D slicer^55^ to place eight 3D anatomical landmarks, common to all specimens, and seven 3D semilandmarks at the edges of the distal humerus articular surface (they started and ended at anatomical landmarks^56^) (SI-Fig. S5).

We used the 7 semilandmarks curves to extract a point cloud of the distal humerus articular surface. Then, we fitted a cylinder surface to the point cloud and determined the maximum diameter, $D_{max}$; minimum diameter, $D_{min}$; and the width, *L*, of the articular surface (SI-Fig. S6) (for the measurements of the distal humerus we developed a code in python published in https://doi.org/10.5281/zenodo.7993776). The radius of the fitted cylinder was defined as the average radius, *D*. As in previous studies, the measurements for species with multiple specimens were averaged^57–59^.

### Phylogenetic analysis

We used theoretical power-law relationships for scaling the parameters to body mass^58,60^: $Y = aX^{b}$, where *Y* and *X* are the related parameters. By $\log _{10}$-transforming both sides, we obtain a linear function: $log_{10}(Y)=\log _{10}(a)+b~\log _{10}(X)$, where $\log _{10}(a)$ is the elevation of the line and *b* the slope.

We used phylogenetic generalized least-squares regressions (PGLS) to consider the impact of evolutionary relatedness on the data, using 10,000 phylogenetic trees obtained from the tool VertLife^61^. To quantify the extent to which the measurements were affected by evolutionary history, we estimated Pagel’s $\lambda$ by maximum likelihood optimization^62,63^. All continuous variables were $\log _{10}$-transformed prior to the statistical analyses. We calculated allometries for all phylogenies and selected an average tree to serve as the representative tree (SI-Fig. S4). The ’ape’ and ’caper’ packages for R^34,64,65^ were used for the analyses.

We also used the standardized major axis (SMA) line-fitting method to examine the scaling relationships over the full range of body mass. While this approach does not consider phylogenetic relationships, provides a useful model for predicting patterns^58^. Similar values for *X* often correspond to similar values for *Y*, but this does not necessarily indicate evolutionary relatedness^66^. We used the ’smatr’ package in R^65,67,68^. The results are presented in SI-Table S5.

### Supplementary Information

Supplementary Information.

## Data Availability

The datasets generated and/or analysed during the current study are available in a Zenodo repository, https://doi.org/10.5281/zenodo.7993776.
