# Supplementary material for: Elbow dimensions in quadrupedal mammals driven by lubrication regime
Source: Sci Rep. 2024 Jan 25;14:2177. doi: 10.1038/s41598-023-50619-x (PMC10810906; doi:10.1038/s41598-023-50619-x)
Supplement: Supplementary file 1 — Supplementary Information. [file 41598_2023_50619_MOESM1_ESM.pdf]

Supplementary Information: Elbow dimensions in quadrupedal mammals driven by lubrication regime

Kalenia Marquez-Florez[a,*], Santiago Arroyave-Tobon[a], Loïc Tadrist[a], and Jean-Marc Linares[a]

[a]Aix Marseille Univ, CNRS, ISM, Marseille, France
[*]Corresponding author: kalenia-maria.marquez-florez@univ-amu.fr

Bones in the study

Table S1: List of the humerus from MorphoSource.org used in the study

Scientific name	NCBI	ARK or DOI	Object	MorphoSourse ID	$D_{max}/2$	$D_{min}/2$	D	L	l
Didelphis virginiana	9267	ark:/87602/m4/399433	dmu:mamm:005	ID 000399433	3.47	1.31	2.61	9.96	69.92
Didelphis marsupialis	9268	ark:/87602/m4/M86079	nhmuk:zoo:1948.7.12.5	ID 000086079	2.68	1.09	2.01	7.51	61.56
Dasyurus hallucatus	9280	ark:/87602/m4/M82293	mnh:mammals:119804	ID 000082293	2.22	0.76	1.51	6.69	39.47
Dasyurus maculatus	9281	ark:/87602/m4/M68242	umzc:vertebrates:a6. 10/3	ID 000068242	3.99	1.81	2.94	10.70	68.79
Antechinus stuartii	9283	ark:/87602/m4/M82265	fmnh:mammals:129556	ID 000082265	0.74	0.31	0.52	2.30	15.14
Sminthopsis crassicaudata	9301	ark:/87602/m4/M82325	fmnh:mammals:72923	ID 000082325	0.65	0.29	0.43	2.12	11.75
Sarcophilus harrisii	9305	ark:/87602/m4/M86127	nhmuk:zoo:2003.331	ID 000086127	6.32	3.26	4.80	19.55	99.44
Sarcophilus harrisii	9305	ark:/87602/m4/447372	SAMA:Mammalogy:M2153	ID 000447372	7.75	3.51	5.72	21.32	109.30
Trichosurus vulpecula	9337	ark:/87602/m4/M68234	mzc:vertebrates:a9.16/7	ID 000068234	4.24	1.15	2.71	15.32	78.06
Canis lupus	9612	ark:/87602/m4/M106364	imnh:r:831	ID 000106364	11.21	5.10	7.43	22.45	141.40
Canis lupus	9612	ark:/87602/m4/M107516	imnh:r:844	ID 000107516	14.90	7.48	10.13	31.23	196.60
Canis lupus	9612	ark:/87602/m4/M25741	ncsu:cvm:31	ID 000025741	14.37	6.64	10.04	29.24	192.60
Canis lupus	9612	ark:/87602/m4/M25754	ncsu:cvm:31	ID 000025754	14.48	7.02	10.19	29.13	194.50
Canis lupus	9612	ark:/87602/m4/M31840	uf:mammals:8379	ID 000031840	9.39	4.75	6.85	20.97	141.80
Canis lupus	9612	ark:/87602/m4/388442	dmu:mamm:003	ID 000388442	12.18	5.78	8.52	26.56	166.20
Canis latrans	9614	ark:/87602/m4/M31861	uf:mammals:13412	ID 000031861	10.73	4.34	7.04	21.67	160.10
Canis latrans	9614	ark:/87602/m4/M106752	imnh:r:396	ID 000106752	9.42	5.28	6.98	20.31	162.20
Canis latrans	9614	ark:/87602/m4/382997	L-rhd	ID 000382997	10.11	5.14	7.13	20.07	157.10
Canis latrans	9614	ark:/87602/m4/388359	mu:mamm:002	ID 000388359	9.91	5.03	7.29	19.46	157.10
Vulpes vulpes	9627	ark:/87602/m4/M31944	uf:mammals:14189	ID 000031944	6.68	3.81	5.07	13.82	126.40
Vulpes vulpes	9627	ark:/87602/m4/M105316	imnh:r:94	ID 000105316	6.71	4.41	5.32	13.16	130.90
Helarctos malayanus	9634	ark:/87602/m4/446974	amnh:mammals:m-35364	ID 000446974	11.53	4.52	8.70	45.63	224.10
Melursus ursinus	9636	ark:/87602/m4/446957	AMNH:Mammals:M-22896	ID 000446957	17.73	8.36	12.14	50.50	229.90
Tremarctos ornatus	9638	ark:/87602/m4/446883	AMNH:Mammals:M-100010	ID 000446883	10.84	3.35	8.35	39.63	209.40
Ursus arctos	9644	ark:/87602/m4/M120221	imnh:r:2400	ID 000120221	16.62	7.57	11.64	62.87	294.10
Ursus arctos	9644	ark:/87602/m4/M120223	imnh:r:2400	ID 000120223	23.24	11.96	17.09	83.07	296.50
Ursus arctos	9644	ark:/87602/m4/M123500	mvz:mammal specimens:4385	ID 000123500	23.24	11.96	17.09	83.07	383.60
Ursus arctos	9644	ark:/87602/m4/427757	UWBM	ID 000427757	26.92	13.06	18.40	90.71	409.50
Procyon lotor	9654	ark:/87602/m4/M110218	imnh:r:909	ID 000110218	7.07	3.34	4.86	16.85	92.04
Procyon lotor	9654	ark:/87602/m4/M110282	imnh:r:909	ID 000110282	6.60	3.63	4.92	17.24	91.25
Felis catus	9685	ark:/87602/m4/M31869	uf:mammals:8614	ID 000031869	4.56	2.22	3.29	11.97	93.00
Felis catus	9685	ark:/87602/m4/382988	L-rhd	ID 000382988	5.99	2.77	4.34	15.33	115.30
Felis catus	9685	ark:/87602/m4/388304	mu:mamm:001	ID 000388304	5.98	2.72	4.33	15.31	115.40
Panthera leo	9689	ark:/87602/m4/M31932	uf:mammals:8428	ID 000031932	20.48	9.88	15.42	60.82	307.80
Panthera pardus	9691	ark:/87602/m4/M119883	imnh:r:2372	ID 000119883	14.15	6.24	9.95	39.82	213.40
Panthera pardus	9691	ark:/87602/m4/M119885	imnh:r:2372	ID 000119885	13.34	6.65	10.11	36.81	207.30
Panthera tigris	9694	ark:/87602/m4/M123256	mvz:mammal specimens:189634	ID 000123256	19.67	7.56	13.59	60.92	305.30
Puma concolor	9696	ark:/87602/m4/M31935	uf:mammals:25908	ID 000031935	13.82	5.83	10.00	37.29	237.80
Puma concolor	9696	ark:/87602/m4/M104608	imnh:r:996	ID 000104608	14.71	5.79	10.40	41.74	239.30
Elephas maximus	9783	ark:/87602/m4/M168766	Imnh:1486	ID 000168766	62.75	35.67	47.63	160.73	797.00
Elephas maximus	9783	ark:/87602/m4/M168768	Imnh:1486	ID 000168768	71.12	37.87	50.92	169.14	797.90
Equus zebra	9791	ark:/87602/m4/M121651	imnh:r:2425	ID 000121651	24.71	15.04	19.56	70.65	251.80
Equus caballus	9796	ark:/87602/m4/M122806	mvz:mammal specimens:162289	ID 000122806	35.06	21.28	26.16	80.77	310.20
Tapirus indicus	9802	ark:/87602/m4/423619	UWBM	ID 000423619	28.17	16.32	21.65	66.12	279.20
Diceros bicornis	9805	ark:/87602/m4/364937	rmca:vert:RG2133	ID 000364937	45.30	17.21	31.93	100.07	379.60
Diceros bicornis	9805	ark:/87602/m4/365776	mnhn:zm:AC-1936-644	ID 000365776	43.27	19.85	30.90	97.51	363.50
Diceros bicornis	9805	ark:/87602/m4/367251	Mdc:50002040	ID 000367251	39.85	18.88	28.10	94.03	348.60
Diceros bicornis	9805	ark:/87602/m4/367352	Mdc:50002046	ID 000367352	46.73	17.68	30.51	97.81	366.50
Diceros bicornis	9805	ark:/87602/m4/421099	amnh:mammals:M-81805	ID 000421099	46.34	22.66	33.35	105.25	400.30
Diceros bicornis	9805	ark:/87602/m4/421149	AMNH:Mammals:M-27757	ID 000421149	46.82	20.51	32.43	114.92	409.00
Diceros bicornis	9805	ark:/87602/m4/421224	AMNH:Mammals:M-113777	ID 000421224	45.68	22.47	32.64	106.15	404.20
Diceros bicornis	9805	ark:/87602/m4/421251	AMNH:Mammals:M-113778	ID 000421251	45.64	20.53	30.94	98.09	380.10
Ceratotherium simum	9807	ark:/87602/m4/365666	mnhn:zm:MO-2005-297	ID 000365666	49.24	22.54	33.87	120.48	407.30
Ceratotherium simum	9807	ark:/87602/m4/421069	amnh:mammals:M-81815	ID 000421069	54.83	24.32	38.66	120.18	462.10
Ceratotherium simum	9807	ark:/87602/m4/364703	p-cm:NH.CON.20	ID 000364703	53.40	23.81	38.34	119.97	391.50
Ceratotherium simum	9807	ark:/87602/m4/364758	p-cm:NH.CON.32	ID 000364758	51.74	21.53	35.60	111.65	368.10
Ceratotherium simum	9807	ark:/87602/m4/364783	p-cm:NH.CON.37	ID 000364783	48.87	21.85	34.33	106.53	383.20
Ceratotherium simum	9807	ark:/87602/m4/364808	p-cm:NH.CON.40	ID 000364808	48.72	20.33	34.56	107.92	379.50
Ceratotherium simum	9807	ark:/87602/m4/364833	p-cm:NH.CON.110	ID 000364833	48.30	23.24	34.26	102.98	392.60
Ceratotherium simum	9807	ark:/87602/m4/364858	p-cm:NH.CON.112	ID 000364858	51.05	20.83	36.05	113.46	406.60
Ceratotherium simum	9807	ark:/87602/m4/364883	rmca:vert:1985.32-M-0001	ID 000364883	47.41	19.71	33.13	102.54	391.40

Continued on next page

Table S1 – *Continued from previous page*

Scientific name	NCBI	ARK or DOI	Object	MorphoSource ID	$D_{max}/2$	$D_{min}/2$	D	L	l
Ceratotherium simum	9807	ark:/87602/m4/364908	rmca:vert:RG35146	ID 000364908	52.95	21.63	37.22	113.78	412.20
Ceratotherium simum	9807	ark:/87602/m4/420905	amnh:mammals:M-51854	ID 000420905	50.42	22.20	35.54	102.05	401.40
Ceratotherium simum	9807	ark:/87602/m4/420938	amnh:mammals:M-51855	ID 000420938	56.52	24.36	39.41	118.53	420.40
Ceratotherium simum	9807	ark:/87602/m4/420972	amnh:mammals:M-51857	ID 000420972	47.13	23.41	35.62	111.73	398.60
Ceratotherium simum	9807	ark:/87602/m4/421003	amnh:mammals:M-51858	ID 000421003	51.49	21.60	35.55	106.21	414.20
Rhinoceros unicornis	9809	ark:/87602/m4/365950	mnhn:zm:AC-1885-734	ID 000365950	52.47	22.53	38.34	114.11	475.80
Rhinoceros unicornis	9809	ark:/87602/m4/365984	mnhn:zm:AC-1932-49	ID 000365984	52.37	26.17	36.78	113.67	463.00
Rhinoceros unicornis	9809	ark:/87602/m4/366038	mnhn:zm:AC-1960-59	ID 000366038	53.92	24.39	38.91	112.78	465.80
Rhinoceros unicornis	9809	ark:/87602/m4/366056	mnhn:zm:AC-1967-101	ID 000366056	49.40	22.81	36.18	110.33	430.60
Rhinoceros unicornis	9809	ark:/87602/m4/421571	AMNH:Mammals:M-35759	ID 000421571	48.32	19.64	35.13	109.76	446.80
Rhinoceros unicornis	9809	ark:/87602/m4/421597	AMNH:Mammals:M-54456	ID 000421597	49.59	22.01	35.28	107.44	431.50
Orycteropus afer	9818	ark:/87602/m4/446927	AMNH:Mammals:M-119504	ID 000446927	11.00	5.15	8.37	35.44	154.40
Camelus dromedarius	9838	ark:/87602/m4/M111892	imnh:r:1007	ID 000111892	35.98	19.47	28.45	80.06	407.60
Lama glama	9844	ark:/87602/m4/M120933	imnh:r:2392	ID 000120933	16.76	7.95	12.81	44.79	279.90
Lama glama	9844	ark:/87602/m4/M120935	imnh:r:2392	ID 000120935	16.59	7.75	12.90	44.64	276.20
Cervus elaphus	9860	ark:/87602/m4/M122050	imnh:r:749	ID 000122050	21.21	12.29	17.90	54.22	245.20
Cervus elaphus	9860	ark:/87602/m4/M122158	imnh:r:749	ID 000122158	21.28	10.17	17.82	52.98	246.70
Odocoileus hemionus	9872	ark:/87602/m4/M105109	imnh:r:146	ID 000105109	14.72	6.82	11.44	37.36	199.30
Odocoileus hemionus	9872	ark:/87602/m4/M105187	imnh:r:146	ID 000105187	14.16	7.42	11.51	37.29	201.70
Odocoileus hemionus	9872	ark:/87602/m4/M122296	imnh:r:2093	ID 000122296	15.63	8.13	12.05	41.59	230.10
Odocoileus virginianus	9874	ark:/87602/m4/388385	L-rhd:02	ID 000388385	14.97	8.41	11.46	37.03	188.00
Antilocapra americana	9891	ark:/87602/m4/M108322	imnh:r:897	ID 000108322	13.14	8.12	10.98	34.67	202.70
Giraffa camelopardalis	9894	ark:/87602/m4/M119583	imnh:r:2283	ID 000119583	38.71	27.13	32.19	112.66	499.10
Giraffa camelopardalis	9894	ark:/87602/m4/M119585	imnh:r:2283	ID 000119585	38.74	27.30	32.08	113.03	500.50
Ammotragus lervia	9899	ark:/87602/m4/399110	MNHN:ZM:2010-643	ID 000399110	13.14	8.46	10.77	42.45	215.70
Ammotragus lervia	9899	ark:/87602/m4/399156	MNHN:ZM:AC-1896-439	ID 000399156	13.80	8.64	10.82	47.24	236.60
Bison bison	9901	ark:/87602/m4/M106144	imnh:r:534	ID 000106144	29.81	18.52	24.49	95.01	378.40
Bison bison	9901	ark:/87602/m4/M106236	imnh:r:534	ID 000106236	29.51	18.05	24.15	93.82	378.40
Bison bison	9901	ark:/87602/m4/398778	MNHN:ZM:AC-1885-339	ID 000398778	26.21	16.92	21.78	74.37	321.20
Bison bison	9901	ark:/87602/m4/398820	MNHN:ZM:AC-1902-316	ID 000398820	26.00	16.91	22.00	84.68	357.60
Bos javanicus	9906	ark:/87602/m4/398872	MNHN:ZM:AC-1944-101	ID 000398872	24.33	16.14	20.11	74.84	313.70
Bos javanicus	9906	ark:/87602/m4/398920	MNHN:ZM:MO-1967-1689	ID 000398920	20.90	13.34	17.38	66.88	281.90
Bos taurus	9913	ark:/87602/m4/398837	NHN:ZM:AC-1926-302	ID 000398837	26.22	16.77	21.46	85.86	328.10
Boselaphus tragocamelus	9917	ark:/87602/m4/398475	MNHN:ZM:AC-1864-103	ID 000398475	21.07	14.06	17.15	53.52	248.10
Boselaphus tragocamelus	9917	ark:/87602/m4/398492	MNHN:ZM:AC-1907-146	ID 000398492	21.44	14.74	17.61	58.06	266.30
Capra hircus	9925	ark:/87602/m4/399077	MNHN:ZM:2007-1349	ID 000399077	11.71	7.24	9.29	31.35	184.00
Connochaetes taurinus	9927	ark:/87602/m4/397792	MNHN:ZM:2013-26	ID 000397792	17.34	11.11	13.88	46.00	202.30
Connochaetes taurinus	9927	ark:/87602/m4/397823	MNHN:ZM:MO-1976-344	ID 000397823	16.57	11.19	13.62	48.66	206.10
Ovis aries	9940	ark:/87602/m4/M106904	imnh:r:862	ID 000106904	14.27	4.27	11.55	42.38	227.40
Ovis aries	9940	ark:/87602/m4/M107004	imnh:r:862	ID 000107004	15.34	9.40	11.69	41.49	228.10
Ovis aries	9940	ark:/87602/m4/399310	MNHN:ZM:AC-2000-438	ID 000399310	11.07	7.72	9.29	32.10	159.20
Taurotragus oryx	9945	ark:/87602/m4/400115	MNHN:ZM:AC-A7983	ID 000400115	29.72	16.79	22.22	74.25	334.20
Oryx gazella	9958	ark:/87602/m4/399838	MNHN:ZM:AC-1905-227	ID 000399838	15.82	10.44	12.72	45.30	194.20
Oryx gazella	9958	ark:/87602/m4/399891	MNHN:ZM:AC-1972-106	ID 000399891	16.43	11.11	13.58	48.98	221.10
Oryx gazella	9958	ark:/87602/m4/399913	MNHN:ZM:AC-1994-9	ID 000399913	18.77	12.46	15.54	55.10	239.20
Kobus ellipsiprymnus	9962	ark:/87602/m4/400043	MNHN:ZM:AC-1935-637	ID 000400043	21.64	12.45	16.67	55.66	238.10
Kobus ellipsiprymnus	9962	ark:/87602/m4/400060	MNHN:ZM:MO-1974-112	ID 000400060	20.85	11.35	16.26	51.23	245.90
Capricornis sp	9966	ark:/87602/m4/399139	MNHN:ZM:AC-1874-283	ID 000399139	20.42	10.14	15.16	50.53	268.70
Syncerus caffer	9970	ark:/87602/m4/398854	MNHN:ZM:AC-1936-72	ID 000398854	26.40	16.47	20.90	80.99	300.20
Marmota monax	9995	DOI: 10.17602/M2/M158256	amnh:mammals:m-67882	ID 000158256	4.66	2.07	3.14	12.67	68.04
Hystrix cristata	10137	ark:/87602/m4/447002	AMNH:Mammals:M-87220	ID 000447002	7.46	3.36	5.68	20.18	110.40
Hydrochoerus hydrochaeris	10149	DOI: 10.17602/M2/M158236	mnh:mammals:m-206440	ID 000158236	13.26	6.06	9.61	28.69	175.20
Myocastor coypus	10157	ark:/87602/m4/M118959	imnh:r:2124	ID 000118959	4.56	2.55	3.52	12.97	67.77
Myocastor coypus	10157	ark:/87602/m4/M119027	imnh:r:2124	ID 000119027	4.47	2.61	3.41	12.60	67.79
Myocastor coypus	10157	DOI: 10.17602/M2/M158266	mnh:mammals:m-206456	ID 000158266	4.65	2.33	3.40	13.31	69.42
Bubalus depressicornis	27596	ark:/87602/m4/398560	MNHN:ZM:2009-421	ID 000398560	14.90	9.39	12.34	45.07	206.40
Bubalus depressicornis	27596	ark:/87602/m4/398733	MNHN:ZM:2009-421	ID 000398733	15.44	8.92	12.46	45.12	207.60
Ursus maritimus	29073	ark:/87602/m4/M169471	mvz:Mammal specimens:125562	ID 000169471	21.19	8.12	14.60	74.63	259.70
Vombatus ursinus	29139	ark:/87602/m4/446918	nmv:mammalogy:6697	ID 000446918	10.39	2.96	7.29	34.20	124.80
Bos frontalis	30520	ark:/87602/m4/398890	MNHN:ZM:AC-1965-120	ID 000398890	25.70	12.54	19.94	80.25	306.60
Bos frontalis	30520	ark:/87602/m4/398903	MNHN:ZM:AC-1970-280	ID 000398903	27.32	14.24	22.23	90.48	321.30
Bos grunniens	30521	ark:/87602/m4/398546	MNHN:ZM:2008-107	ID 000398546	25.75	16.97	20.38	81.47	319.00
Bos grunniens	30521	ark:/87602/m4/398803	MNHN:ZM:AC-1886-300	ID 000398803	21.21	13.77	17.38	67.29	286.70
Taxidea taxus	30554	ark:/87602/m4/M106526	imnh:r:230	ID 000106526	7.00	3.45	5.49	21.57	101.50
Taxidea taxus	30554	ark:/87602/m4/M106542	imnh:r:230	ID 000106542	7.34	3.69	5.61	21.09	101.20

Continued on next page

Table S1 – *Continued from previous page*

Scientific name	NCBI	ARK or DOI	Object	MorphoSourse ID	$D_{max}/2$	$D_{min}/2$	D	L	l
Lasiorhinus latifrons	30668	ark:/87602/m4/446905	nmv:c:22340	ID 000446905	8.19	3.24	6.51	32.71	111.70
Canis simensis	32534	DOI: 10.17602/M2/M158186	mnh:mammals:m-81001	ID 000158186	11.57	5.59	7.63	21.85	185.10
Acinonyx jubatus	32536	ark:/87602/m4/M31645	uf:mammals:14386	ID 000031645	10.95	5.27	8.03	26.71	218.10
Acinonyx jubatus	32536	DOI: 10.17602/M2/M158176	amnh:mammals:m-119657	ID 000158176	12.36	5.06	8.73	30.79	241.90
Leopardus pardalis	32538	ark:/87602/m4/M31875	uf:mammals:4644	ID 000031875	8.39	4.25	6.08	22.44	153.60
Erethizon dorsatum	34844	ark:/87602/m4/M106602	imnh:r:822	ID 000106602	7.54	4.10	5.33	18.46	92.81
Rupicapra rupicapra	34869	ark:/87602/m4/399173	MNHN:ZM:AC-1923-2326	ID 000399173	10.18	5.70	8.01	25.92	165.00
Rupicapra rupicapra	34869	ark:/87602/m4/399297	MNHN:ZM:AC-1995-183	ID 000399297	10.64	7.12	8.88	28.08	174.90
Naemorhedus goral	34871	ark:/87602/m4/399203	MNHN:ZM:AC-1963-320	ID 000399203	9.75	5.54	8.03	28.07	164.70
Oreamnos americanus	34873	ark:/87602/m4/M119273	imnh:r:2352	ID 000119273	13.69	8.32	10.59	40.53	206.00
Oreamnos americanus	34873	ark:/87602/m4/M119367	imnh:r:2352	ID 000119367	13.18	8.48	10.74	36.83	206.00
Oreamnos americanus	34873	ark:/87602/m4/399095	MNHN:ZM:2009-253	ID 000399095	17.97	10.20	13.54	48.94	266.60
Saiga tatarica	34875	ark:/87602/m4/398090	MNHN:ZM:AC-1959-177	ID 000398090	10.98	7.07	8.66	25.70	139.30
Saiga tatarica	34875	ark:/87602/m4/398117	MNHN:ZM:AC-1964-313	ID 000398117	10.86	7.11	8.59	27.46	145.20
Ovibos moschatus	37176	ark:/87602/m4/M121146	imnh:r:2515	ID 000121146	22.96	13.10	17.86	63.85	302.60
Ovibos moschatus	37176	ark:/87602/m4/399281	MNHN:ZM:AC-1977-43	ID 000399281	24.00	14.10	19.23	64.62	295.20
Hemitragus jemlahicus	37179	ark:/87602/m4/M119750	imnh:r:2261	ID 000119750	14.43	8.13	11.23	43.00	239.30
Hemitragus jemlahicus	37179	ark:/87602/m4/399242	MNHN:ZM:AC-1972-133	ID 000399242	13.37	7.29	10.03	34.71	186.80
Hippotragus equinus	37186	ark:/87602/m4/399857	MNHN:ZM:AC-1969-167	ID 000399857	18.88	12.71	15.35	56.25	250.80
Hippotragus equinus	37186	ark:/87602/m4/399934	MNHN:ZM:AC-1995-147	ID 000399934	20.64	13.30	17.13	59.23	261.60
Gazella dorcas	37751	ark:/87602/m4/398243	MNHN:ZM:MO-1968-803	ID 000398243	9.45	6.14	7.65	20.91	128.30
Gazella dorcas	37751	ark:/87602/m4/398255	MNHN:ZM:MO-1974-113	ID 000398255	8.21	5.29	6.61	20.84	118.30
Oryx leucoryx	39411	ark:/87602/m4/399965	MNHN:ZM:MO-1996-2100	ID 000399965	15.37	10.41	12.27	43.15	196.30
Oryx leucoryx	39411	ark:/87602/m4/399982	MNHN:ZM:MO-1996-2101	ID 000399982	14.20	9.61	11.72	42.85	191.00
Gulo gulo	48420	ark:/87602/m4/M121302	imnh:r:2671	ID 000121302	8.42	5.08	6.73	29.37	139.80
Gulo gulo	48420	ark:/87602/m4/446966	AMNH:Mammals:M-35081	ID 000446966	8.26	4.46	6.07	24.85	124.90
Cephalophus silvicultor	50347	ark:/87602/m4/399816	MNHN:ZM:MO-1981-1023	ID 000399816	12.62	8.76	10.83	33.65	198.20
Castor canadensis	51338	DOI: 10.17602/M2/M158206	amnh:mammals:m-150136	ID 000158206	5.30	2.35	4.03	18.36	84.00
Myrmecobius fasciatus	55782	ark:/87602/m4/M68250	umzc:vertebrates:a6. 41/8	ID 000068250	1.86	0.78	1.39	6.55	30.94
Tapirus bairdii	56117	ark:/87602/m4/M58226	amnh:mammals:90128	ID 000058226	22.72	10.60	15.98	55.71	240.80
Tapirus bairdii	56117	ark:/87602/m4/M169248	mvz:Mammal specimens:141173	ID 000169248	21.67	12.15	16.81	52.82	251.20
Addax nasomaculatus	59515	ark:/87602/m4/399874	MNHN:ZM:AC-1970-277	ID 000399874	16.37	10.66	13.18	50.94	204.40
Alcelaphus buselaphus	59517	ark:/87602/m4/397805	MNHN:ZM:AC-1899-238	ID 000397805	15.52	9.67	12.59	42.59	197.20
Antidorcas marsupialis	59523	ark:/87602/m4/398134	MNHN:ZM:AC-1971-89	ID 000398134	12.32	8.13	10.01	31.41	154.70
Antidorcas marsupialis	59523	ark:/87602/m4/398289	MNHN:ZM:MO-1993-1670	ID 000398289	10.07	6.26	8.43	25.52	127.50
Antilope cervicapra	59525	ark:/87602/m4/397840	MNHN:ZM:AC-1901-174	ID 000397840	11.43	6.54	8.93	30.18	151.80
Antilope cervicapra	59525	ark:/87602/m4/398275	MNHN:ZM:MO-1992-618	ID 000398275	10.37	5.85	8.44	28.89	145.70
Ourebia ourebi	59536	ark:/87602/m4/398152	MNHN:ZM:AC-1972-93	ID 000398152	8.85	5.39	7.25	20.58	116.70
Pseudois nayaur	59542	ark:/87602/m4/399228	MNHN:ZM:AC-1966-136	ID 000399228	11.83	7.61	9.90	34.89	200.50
Pseudois nayaur	59542	ark:/87602/m4/399259	MNHN:ZM:AC-1972-92	ID 000399259	11.82	7.03	9.64	36.19	195.00
Redunca arundinum	59554	ark:/87602/m4/400013	MNHN:ZM:AC-1881-1147	ID 000400013	10.72	5.24	8.37	25.68	140.30
Redunca arundinum	59554	ark:/87602/m4/400030	MNHN:ZM:AC-1923-2173	ID 000400030	10.80	5.59	8.56	27.18	135.70
Felis margarita	61378	ark:/87602/m4/M110522	imnh:r:938	ID 000110522	4.30	1.73	3.21	12.17	98.43
Felis margarita	61378	ark:/87602/m4/M110540	imnh:r:938	ID 000110540	4.49	1.75	3.26	11.93	99.10
Lynx rufus	61384	ark:/87602/m4/M31892	uf:mammals:24023	ID 000031892	6.98	3.11	5.27	19.40	155.40
Caracal caracal	61394	DOI: 10.17602/M2/M158196	amnh:mammals:m-113794	ID 000158196	6.47	2.74	4.83	17.71	128.90
Leptailurus serval	61405	ark:/87602/m4/M166839	mnhn:zm:ac-1963-75	ID 000166839	8.42	2.96	6.06	22.35	187.10
Neofelis nebulosa	61452	ark:/87602/m4/M31925	uf:mammals:10771	ID 000031925	9.12	3.55	6.47	28.77	162.50
Echymipera rufescens	65611	ark:/87602/m4/M82302	fmnh:mammals:60525	ID 000082302	2.68	1.05	1.91	7.82	45.20
Isoodon obesulus	65612	ark:/87602/m4/M82443	umzc:vertebrates:a7. 4/5	ID 000082443	2.26	0.93	1.60	6.11	37.58
Tragelaphus scriptus	66440	ark:/87602/m4/400084	MNHN:ZM:AC-1980-7	ID 000400084	12.39	6.32	9.63	32.60	191.90
Oreotragus oreotragus	66444	ark:/87602/m4/399999	MNHN:ZM:2007-1388	ID 000399999	7.26	3.73	5.73	18.35	116.70
Speothos venaticus	68741	ark:/87602/m4/M31941	uf:mammals:19126	ID 000031941	6.65	3.23	4.87	17.38	103.10
Eudorcas thomsonii	69308	ark:/87602/m4/398104	MNHN:ZM:AC-1961-41	ID 000398104	8.88	5.10	7.24	22.12	122.60
Eudorcas thomsonii	69308	ark:/87602/m4/398224	MNHN:ZM:MO-1962-384	ID 000398224	10.06	5.55	7.97	23.95	128.90
Tupaia tana	70687	ark:/87602/m4/M34008	fmnh:mammals:145465	ID 000034008	1.49	0.84	1.19	4.84	32.75
Tetracerus quadricornis	73823	ark:/87602/m4/398511	MNHN:ZM:MO-1988-223	ID 000398511	8.20	4.79	6.41	19.15	114.90
Tetracerus quadricornis	73823	ark:/87602/m4/398528	MNHN:ZM:MO-1993-4627	ID 000398528	8.07	5.45	6.71	20.97	129.30
Dicerorhinus sumatrensis	89632	ark:/87602/m4/M169046	mvz:Mammal specimens:208920	ID 000169046	35.42	18.24	24.87	73.26	354.10
Dicerorhinus sumatrensis	89632	ark:/87602/m4/365710	mnhn:zm:AC-1903-300	ID 000365710	38.22	18.24	27.01	80.20	364.00
Dicerorhinus sumatrensis	89632	ark:/87602/m4/365751	mnhn:zm:AC-A7967	ID 000365751	33.90	18.61	25.31	68.18	354.20
Genetta genetta	94190	ark:/87602/m4/M166845	mnhn:zm:mo-1997-450	ID 000166845	3.52	1.86	2.71	10.21	73.65
Rhinoceros sondaicus	102233	ark:/87602/m4/365850	mnhn:zm:AC-A7970	ID 000365850	48.58	23.53	35.29	102.38	455.40
Rhinoceros sondaicus	102233	ark:/87602/m4/365897	mnhn:zm:AC-A7971	ID 000365897	49.02	25.27	35.67	102.55	430.50
Rhinoceros sondaicus	102233	ark:/87602/m4/367406	Mdc:50002041	ID 000367406	44.46	21.19	32.51	102.10	456.30

Continued on next page

Table S1 – *Continued from previous page*

**Scientific name**	**NCBI**	**ARK or DOI**	**Object**	**MorphoSourse ID**	$D_{max}/2$	$D_{min}/2$	D	L	l
Caluromysiops irrupta	126286	ark:/87602/m4/M82284	fmnh:mammals:60698	ID 000082284	1.96	0.71	1.35	8.29	38.58
Tupaia minor	143289	ark:/87602/m4/M32449	fmnh:mammals:76865	ID 000032449	0.93	0.52	0.70	2.69	21.55
Tupaia minor	143289	ark:/87602/m4/M33722	fmnh:mammals:76865	ID 000033722	0.91	0.51	0.70	2.73	21.58
Tolypeutes matacus	183749	DOI: 10.17602/M2/M158276	amnh:mammals:m-248394	ID 000158276	2.76	1.18	2.02	10.00	44.68
Acrobates pygmaeus	190720	ark:/87602/m4/M52804	nhmuk:zoo:82.7.29.23	ID 000052804	0.57	0.28	0.44	2.28	12.67
Urogale everetti	192726	ark:/87602/m4/M34160	fmnh:mammals:57312	ID 000034160	1.71	0.83	1.31	5.27	32.24
Urogale everetti	192726	ark:/87602/m4/M34164	fmnh:mammals:57312	ID 000034164	1.66	0.74	1.28	5.29	32.35
Ptilocercus lowii	320338	ark:/87602/m4/M96392	fmnh:mammals:57450	ID 000096392	1.06	0.49	0.73	3.65	22.12
Tupaia palawanensis	320359	ark:/87602/m4/M36015	fmnh:mammals:62976	ID 000036015	1.26	0.63	0.95	3.71	27.25
Tupaia palawanensis	320359	ark:/87602/m4/M36041	fmnh:mammals:62976	ID 000036041	1.24	0.62	0.94	3.82	27.39
Hemibelideus lemuroides	596256	ark:/87602/m4/M68192	fmnh:mammals:60926	ID 000068192	2.47	0.90	1.67	9.29	50.00
Geocapromys brownii	1543402	DOI: 10.17602/M2/M158226	amnh:mammals:m-45156	ID 000158226	3.28	1.90	2.56	10.01	55.52

- l: the length of the humerus in mm.
- L: the width of the distal articular surface of the humerus in mm.
- D: average radius of the distal articular surface of the humerus in mm.
- $D_{max}/2$: maximum radius of the distal articular surface of the humerus in mm.
- $D_{min}/2$: minimum radius of the distal articular surface of the humerus in mm.
- NCBI: unique numerical identifier assigned in the National Center for Biotechnology Information (NCBI) Taxonomy database.

Species considered in the study

Table S2: Species used in this study

NCBI	Scientific name	Common name	Order	Family	Mass (Kg)	ref. mass	# bones	l	$D_{max}/2$	$D_{min}/2$	D	L
9267	Didelphis virginiana	North American opossum	Didelphimorphia	Didelphidae	5.00	[30]	1	69.92	3.47	1.31	2.61	9.96
9268	Didelphis marsupialis	Southern opossum	Didelphimorphia	Didelphidae	1.53	[85]	1	61.56	2.68	1.09	2.01	7.51
9280	Dasyurus hallucatus	Northern quoll	Dasyuromorphia	Dasyuridae	0.60	[56]	1	39.47	2.22	0.76	1.51	6.69
9281	Dasyurus maculatus	Spotted-tailed quoll	Dasyuromorphia	Dasyuridae	4.40	[87]	1	68.79	3.99	1.81	2.94	10.70
9283	Antechinus stuartii	Brown antechinus	Dasyuromorphia	Dasyuridae	0.03	[19]	1	15.14	0.74	0.31	0.52	2.30
9301	Sminthopsis crassicaudata	Fat-tailed dunnart	Dasyuromorphia	Dasyuridae	0.02	[19]	1	11.75	0.65	0.29	0.43	2.12
9305	Sarcophilus harrisii	Tasmanian devil	Dasyuromorphia	Dasyuridae	8.00	[66]	2	104.37	7.04	3.38	5.26	20.44
9337	Trichosurus vulpecula	Common brushtail	Diprotodontia	Phalangeridae	4.30	[6]	1	78.06	4.24	1.15	2.71	15.32
9612	Canis lupus	Gray wolf	Carnivora	Canidae	49.00	[22]	2	169.00	13.06	6.29	8.78	26.84
9614	Canis latrans	Coyote	Carnivora	Canidae	12.30	[5]	4	159.13	10.04	4.95	7.11	20.38
9627	Vulpes vulpes	Red fox	Carnivora	Canidae	5.50	[5]	2	128.65	6.70	4.11	5.20	13.49
9634	Helarctos malayanus	Malayan sun bear	Carnivora	Ursidae	55.00	[32]	1	224.10	11.53	4.52	8.70	45.63
9636	Melursus ursinus	Sloth bear	Carnivora	Ursidae	97.50	[55]	1	229.90	17.73	8.36	12.14	50.50
9638	Tremarctos ornatus	Spectacled bear	Carnivora	Ursidae	70.00	[26]	1	209.40	10.84	3.35	8.35	39.63
9644	Ursus arctos	Brown bear	Carnivora	Ursidae	251.30	[19]	4	345.93	22.50	11.14	16.06	79.93
9654	Procyon lotor	Raccoon	Carnivora	Procyonidae	7.50	[5]	2	91.65	6.83	3.49	4.89	17.04
9685	Felis catus	Domestic cat	Carnivora	Felidae	4.75	[4]	3	107.90	5.51	2.57	3.99	14.21
9689	Panthera leo	Lion	Carnivora	Felidae	188.00	[11]	1	307.80	20.48	9.88	15.42	60.82
9691	Panthera pardus	Leopard	Carnivora	Felidae	52.40	[19]	2	210.35	13.74	6.45	10.03	38.32
9694	Panthera tigris	Tiger	Carnivora	Felidae	179.00	[20]	1	305.30	19.67	7.56	13.59	60.92
9696	Puma concolor	Puma	Carnivora	Felidae	50.00	[7]	2	238.55	14.27	5.81	10.20	39.52
9783	Elephas maximus	Asiatic elephant	Proboscidea	Elephantidae	4000.00	[70]	2	797.45	66.94	36.77	49.28	164.93
9791	Equus zebra	Mountain zebra	Perissodactyla	Equidae	300.00	[8]	1	251.80	24.71	15.04	19.56	70.65
9796	Equus caballus	Horse	Perissodactyla	Equidae	529.66	[7]	1	310.20	35.06	21.28	26.16	80.77
9802	Tapirus indicus	Asiatic tapir	Perissodactyla	Tapiridae	317.00	[5]	1	279.20	28.17	16.32	21.65	66.12
9805	Diceros bicornis	Black rhinoceros	Perissodactyla	Rhinocerotidae	1400.00	[8]	8	381.48	44.95	19.97	31.35	101.73
9807	Ceratotherium simum	White rhinoceros	Perissodactyla	Rhinocerotidae	2300.00	[63]	14	407.30	49.24	22.54	33.87	120.48
9809	Rhinoceros unicornis	Greater Indian rhinoceros	Perissodactyla	Rhinocerotidae	1865.50	[15]	6	452.25	51.01	22.93	36.77	111.35
9818	Orycteropus afer	Aardvark	Tubulidentata	Orycteropodidae	61.00	[81]	1	154.40	11.00	5.15	8.37	35.44
9838	Camelus dromedarius	Arabian camel	Artiodactyla	Camelidae	500.00	[14]	1	407.60	35.98	19.47	28.45	80.06
9844	Lama glama	Llama	Artiodactyla	Camelidae	165.00	[36]	2	278.05	16.67	7.85	12.86	44.71
9860	Cervus elaphus	Red deer	Artiodactyla	Cervidae	300.00	[8]	2	245.95	21.25	11.23	17.86	53.60
9872	Odocoileus hemionus	Mule deer	Artiodactyla	Cervidae	96.50	[75]	3	210.37	14.84	7.46	11.67	38.75
9874	Odocoileus virginianus	White-tailed deer	Artiodactyla	Cervidae	97.00	[61]	1	188.00	14.97	8.41	11.46	37.03
9891	Antilocapra americana	Pronghorn	Artiodactyla	Antilocapridae	53.50	[23]	1	202.70	13.14	8.12	10.98	34.67
9894	Giraffa camelopardalis	Giraffe	Artiodactyla	Giraffidae	1555.00	[71]	2	499.80	38.72	27.22	32.14	112.84
9899	Ammotragus lervia	Aoudad	Artiodactyla	Bovidae	104.25	[9]	2	226.15	13.47	8.55	10.79	44.85
9901	Bison bison	American bison	Artiodactyla	Bovidae	612.50	[21]	4	358.90	27.88	17.60	23.10	86.97
9906	Bos javanicus	Banteng	Artiodactyla	Bovidae	700.00	[82]	2	297.80	22.62	14.74	18.75	70.86
9913	Bos taurus	Cattle	Artiodactyla	Bovidae	755.00	[76]	1	328.10	26.22	16.77	21.46	85.86
9917	Boselaphus tragocamelus	Nilgai	Artiodactyla	Bovidae	228.50	[17]	2	257.20	21.26	14.40	17.38	55.79
9925	Capra hircus	Goat	Artiodactyla	Bovidae	45.00	[73]	1	184.00	11.71	7.24	9.29	31.35
9927	Connochaetes taurinus	Brindled gnu	Artiodactyla	Bovidae	179.89	[7]	2	204.20	16.96	11.15	13.75	47.33
9940	Ovis aries	Sheep	Artiodactyla	Bovidae	22.65	[8]	3	204.90	13.56	7.13	10.84	38.66
9945	Tragelaphus oryx	Eland	Artiodactyla	Bovidae	629.50	[24]	1	334.20	29.72	16.79	22.22	74.25
9958	Oryx gazella	Gemsbok	Artiodactyla	Bovidae	215.00	[41]	3	218.17	17.00	11.34	13.95	49.80
9962	Kobus ellipsiprymnus	Waterbuck	Artiodactyla	Bovidae	230.00	[35]	2	242.00	21.24	11.90	16.46	53.45
9966	Capricornis crispus	Japanese serow	Artiodactyla	Bovidae	87.50	[29]	1	268.70	20.42	10.14	15.16	50.53
9970	Syncerus caffer	African buffalo	Artiodactyla	Bovidae	620.00	[19]	1	300.20	26.40	16.47	20.90	80.99
9995	Marmota monax	Woodchuck	Rodentia	Sciuridae	4.00	[19]	1	68.04	4.66	2.07	3.14	12.67
10137	Hystrix cristata	Crested porcupine	Rodentia	Hystricidae	20.00	[72]	1	110.40	7.46	3.36	5.68	20.18
10149	Hydrochoerus hydrochaeris	Capybara	Rodentia	Hydrochaeridae	50.50	[19]	1	175.20	13.26	6.06	9.61	28.69
10157	Myocastor coypus	Nutria	Rodentia	Myocastoridae	7.50	[60]	3	68.33	4.56	2.50	3.44	12.96
27596	Bubalus depressicornis	Lowland anoa	Artiodactyla	Bovidae	157.50	[74]	2	207.00	15.17	9.15	12.40	45.10
29073	Ursus maritimus	Polar bear	Carnivora	Ursidae	265.00	[19]	1	259.70	21.19	8.12	14.60	74.63
29139	Vombatus ursinus	Common wombat	Diprotodontia	Vombatidae	31.00	[48]	1	124.80	10.39	2.96	7.29	34.20
30520	Bos frontalis	Gayal	Artiodactyla	Bovidae	765.50	[1]	2	313.95	26.51	13.39	21.09	85.37
30521	Bos grunniens	Domestic yak	Artiodactyla	Bovidae	650.00	[78]	2	302.85	23.48	15.37	18.88	74.38
30554	Taxidea taxus	American badger	Carnivora	Mustelidae	8.00	[83]	2	101.35	7.17	3.57	5.55	21.33
30668	Lasiorhinus latifrons	Southern hairy-nosed wombat	Diprotodontia	Vombatidae	26.75	[37]	1	111.70	8.19	3.24	6.51	32.71
32534	Canis simensis	Ethiopian wolf	Carnivora	Canidae	15.25	[57]	1	185.10	11.57	5.59	7.63	21.85

Continued on next page

Table S2 – *Continued from previous page*

NCBI	Scientific name	Common name	Order	Family	Mass	ref. mass	# bones	l	$D_{max}/2$	$D_{min}/2$	D	L
32536	Acinonyx jubatus	Cheetah	Carnivora	Felidae	39.00	[7]	2	230.00	11.65	5.17	8.38	28.75
32538	Leopardus pardalis	Ocelot	Carnivora	Felidae	11.05	[38]	1	153.60	8.39	4.25	6.08	22.44
34844	Erethizon dorsatum	North American porcupine	Rodentia	Erethizontidae	9.50	[88]	1	92.81	7.54	4.10	5.33	18.46
34869	Rupicapra rupicapra	Chamois	Artiodactyla	Bovidae	38.02	[7]	2	169.95	10.41	6.41	8.45	27.00
34871	Naemorhedus goral	Na	Artiodactyla	Bovidae	27.50	[59]	1	164.70	9.75	5.54	8.03	28.07
34873	Oreamnos americanus	Mountain goat	Artiodactyla	Bovidae	87.50	[40]	3	226.20	14.95	9.00	11.63	42.10
34875	Saiga tatarica	Na	Artiodactyla	Bovidae	35.00	[8]	2	142.25	10.92	7.09	8.62	26.58
37176	Ovibos moschatus	Muskox	Artiodactyla	Bovidae	290.00	[64]	2	298.90	23.48	13.60	18.54	64.24
37179	Hemitragus jemlahicus	Himalayan tahr	Artiodactyla	Bovidae	89.50	[33]	2	213.05	13.90	7.71	10.63	38.85
37186	Hippotragus equinus	Roan antelope	Artiodactyla	Bovidae	257.50	[34]	2	256.20	19.76	13.01	16.24	57.74
37751	Gazella dorcas	Dorcas gazelle	Artiodactyla	Bovidae	16.50	[28]	2	123.30	8.83	5.71	7.13	20.87
39411	Oryx leucoryx	Arabian oryx	Artiodactyla	Bovidae	161.00	[16]	2	193.65	14.78	10.01	12.00	43.00
48420	Gulo gulo	Wolverine	Carnivora	Mustelidae	12.00	[49]	2	132.35	8.34	4.77	6.40	27.11
50347	Cephalophus silvicultor	Yellow-backed duiker	Artiodactyla	Bovidae	62.50	[62]	1	198.20	12.62	8.76	10.83	33.65
51338	Castor canadensis	American beaver	Rodentia	Castoridae	21.50	[50]	1	84.00	5.30	2.35	4.03	18.36
55782	Myrmecobius fasciatus	Numbat	Dasyuromorphia	Myrmecobiidae	0.48	[19]	1	30.94	1.86	0.78	1.39	6.55
56117	Tapirus bairdii	Baird's tapir	Perissodactyla	Tapiridae	225.00	[89]	2	246.00	22.20	11.38	16.40	54.27
59515	Addax nasomaculatus	Na	Artiodactyla	Bovidae	92.50	[13]	1	204.40	16.37	10.66	13.18	50.94
59517	Alcelaphus buselaphus	Hartebeest	Artiodactyla	Bovidae	149.97	[7]	1	197.20	15.52	9.67	12.59	42.59
59523	Antidorcas marsupialis	Springbok	Artiodactyla	Bovidae	39.50	[54]	2	141.10	11.19	7.20	9.22	28.47
59525	Antilope cervicapra	Blackbuck	Artiodactyla	Bovidae	37.50	[77]	2	148.75	10.90	6.19	8.68	29.54
59536	Ourebia ourebi	Oribi	Artiodactyla	Bovidae	14.49	[7]	1	116.70	8.85	5.39	7.25	20.58
59542	Pseudois nayaur	Bharal	Artiodactyla	Bovidae	55.00	[27]	2	197.75	11.83	7.32	9.77	35.54
59554	Redunca arundinum	Southern reedbuck	Artiodactyla	Bovidae	67.00	[43]	2	138.00	10.76	5.42	8.46	26.43
61378	Felis margarita	Sand cat	Carnivora	Felidae	2.40	[6]	2	98.77	4.40	1.74	3.23	12.05
61384	Lynx rufus	Bobcat	Carnivora	Felidae	9.55	[39]	1	155.40	6.98	3.11	5.27	19.40
61394	Caracal caracal	Na	Carnivora	Felidae	13.90	[25]	1	128.90	6.47	2.74	4.83	17.71
61405	Leptailurus serval	Serval	Carnivora	Felidae	13.50	[58]	1	187.10	8.42	2.96	6.06	22.35
61452	Neofelis nebulosa	Clouded leopard	Carnivora	Felidae	17.00	[68]	1	162.50	9.12	3.55	6.47	28.77
65611	Echymipera rufescens	Long-nosed echymipera	Peramelemorphia	Peroryctidae	1.37	[67]	1	45.20	2.68	1.05	1.91	7.82
65612	Isoodon obesulus	Southern brown bandicoot	Peramelemorphia	Peramelidae	0.72	[8]	1	37.58	2.26	0.93	1.60	6.11
66440	Tragelaphus scriptus	Bushbuck	Artiodactyla	Bovidae	52.00	[45]	1	191.90	12.39	6.32	9.63	32.60
66444	Oreotragus oreotragus	Klipspringer	Artiodactyla	Bovidae	13.00	[65]	1	116.70	7.26	3.73	5.73	18.35
68741	Speothos venaticus	Bush dog	Carnivora	Canidae	5.50	[2]	1	103.10	6.65	3.23	4.87	17.38
69308	Eudorcas thomsonii	Thomson's gazelle	Artiodactyla	Bovidae	25.00	[52]	2	125.75	9.47	5.32	7.61	23.03
70687	Tupaia tana	Large tree shrew	Scandentia	Tupaiidae	0.25	[47]	1	32.75	1.49	0.84	1.19	4.84
73823	Tetracerus quadricornis	Four-horned antelope	Artiodactyla	Bovidae	19.50	[18]	2	122.10	8.13	5.12	6.56	20.06
89632	Dicerorhinus sumatrensis	Sumatran rhinoceros	Perissodactyla	Rhinocerotidae	1400.00	[86]	3	357.43	35.85	18.36	25.73	73.88
94190	Genetta genetta	Small-spotted genet	Carnivora	Viverridae	3.70	[31]	1	73.65	3.52	1.86	2.71	10.21
102233	Rhinoceros sondaicus	Javan rhinoceros	Perissodactyla	Rhinocerotidae	1350.00	[10]	3	447.40	47.35	23.33	34.49	102.34
126286	Caluromysiops irrupta	Black-shouldered opossum	Didelphimorphia	Didelphidae	0.45	[84]	1	38.58	1.96	0.71	1.35	8.29
143289	Tupaia minor	Na	Scandentia	Tupaiidae	0.05	[46]	2	21.57	0.92	0.51	0.70	2.71
183749	Tolypeutes matacus	Na	Cingulata	Chlamyphoridae	1.50	[44]	1	44.68	2.76	1.18	2.02	10.00
190720	Acrobates pygmaeus	Na	Diprotodontia	Acrobatidae	0.01	[19]	1	12.67	0.57	0.28	0.44	2.28
192726	Urogale everetti	Na	Scandentia	Tupaiidae	0.35	[79]	2	32.30	1.68	0.79	1.29	5.28
320338	Ptilocercus lowii	Pen-tailed tree shrew	Scandentia	Tupaiidae	0.06	[42]	1	22.12	1.06	0.49	0.73	3.65
320359	Tupaia palawanensis	Palawan tree shrew	Scandentia	Tupaiidae	0.18	[53]	2	27.32	1.25	0.63	0.94	3.77
596256	Hemibelideus lemuroides	Lemur-like ringtail	Diprotodontia	Pseudocheiridae	0.95	[69]	1	50.00	2.47	0.90	1.67	9.29
1543402	Geocapromys brownii	Jamaician hutia	Rodentia	Capromyidae	1.50	[80]	1	55.52	3.28	1.90	2.56	10.01

- l: length of the humerus in mm.
- L: width of the distal articular surface of the humerus in mm.
- D: average radius of the distal articular surface of the humerus in mm.
- $D_{max}/2$: maximum radius of the distal articular surface of the humerus in mm.
- $D_{min}/2$: minimum radius of the distal articular surface of the humerus in mm.
- NCBI: unique numerical identifier assigned in the National Center for Biotechnology Information (NCBI) Taxonomy database.

Mass references

Peer reviewed

[1] Farshid S Ahrestani. Bos frontalis and Bos gaurus (Artiodactyla: Bovidae). *Mammalian Species*, 50(959):34–50, 08 2018. arXiv:https://academic.oup.com/mspecies/article-pdf/50/959/34/25502514/sey004.pdf, doi:10.1093/mspecies/sey004.

[2] Beatriz de Mello Beisiegel and Gerald L. Zuercher. Speothos venaticus. *Mammalian Species*, (783):1–6, 12 2005. arXiv:https://academic.oup.com/mspecies/article-pdf/doi/10.1644/783.1/8071746/783-1.pdf, doi:10.1644/783.1.

[3] Per Christiansen. Scaling of the limb long bones to body mass in terrestrial mammals. *Journal of Morphology*, 239(2):167–190, 1999. doi:10.1002/(SICI)1097-4687(199902)239:2<167::AID-JMOR5>3.0.CO;2-8.

[4] Per Christiansen. Locomotion in terrestrial mammals: the influence of body mass, limb length and bone proportions on speed. *Zoological Journal of the Linnean Society*, 136(4):685–714, dec 2002. URL: https://academic.oup.com/zoolinnean/article-lookup/doi/10.1046/j.1096-3642.2002.00041.x, doi:10.1046/j.1096-3642.2002.00041.x.

[5] Per Christiansen. Mass allometry of the appendicular skeleton in terrestrial mammals. *Journal of Morphology*, 251(2):195–209, 2002. doi:10.1002/jmor.1083.

[6] F. Russell Cole and Don E. Wilson. Felis margarita (Carnivora: Felidae). *Mammalian Species*, 47(924):63–77, 08 2015. arXiv:https://academic.oup.com/mspecies/article-pdf/47/924/63/8129575/sev007.pdf, doi:10.1093/mspecies/sev007.

[7] Mary Jean P. Currier. Felis concolor. *Mammalian Species*, (200):1–7, 04 1983. arXiv:https://academic.oup.com/mspecies/article-pdf/doi/10.2307/3503951/8070935/200-1.pdf, doi:10.2307/3503951.

[8] Theodore Garland. The relation between maximal running speed and body mass in terrestrial mammals. *Journal of Zoology*, 199(2):157–170, 1983. doi:10.1111/j.1469-7998.1983.tb02087.x.

[9] Gary G. Gray and C. David Simpson. Ammotragus lervia. *Mammalian Species*, (144):1–7, 11 1980. arXiv:https://academic.oup.com/mspecies/article-pdf/doi/10.2307/3504009/24460752/144.pdf, doi:10.2307/3504009.

[10] Colin P. Groves and Jr. Leslie, David M. Rhinoceros sondaicus (Perissodactyla: Rhinocerotidae). *Mammalian Species*, 43(887):190–208, 09 2011. arXiv:https://academic.oup.com/mspecies/article-pdf/43/887/190/8269161/43-887-190.pdf, doi:10.1644/887.1.

[11] Sarah K. Haas, Virginia Hayssen, and Paul R. Krausman. Panthera leo. *Mammalian Species*, (762):1–11, 07 2005. arXiv:https://academic.oup.com/mspecies/article-pdf/doi/10.2307/3504539/8071713/762-1.pdf, doi:10.2307/3504539.

[12] José Iriarte-Díaz. Differential scaling of locomotor performance in small and large terrestrial mammals. *Journal of Experimental Biology*, 205(18):2897–2908, 2002. doi:10.1242/jeb.205.18.2897.

[13] Paul R. Krausman and Anne L. Casey. Addax nasomaculatus. *Mammalian Species*, (807):1–4, 11 2007. arXiv:https://academic.oup.com/mspecies/article-pdf/doi/10.1644/807.1/17749263/807-1.pdf, doi:10.1644/807.1.

[14] Ilse U. Köhler-Rollefson. Camelus dromedarius. *Mammalian Species*, (375):1–8, 04 1991. arXiv:https://academic.oup.com/mspecies/article-pdf/doi/10.2307/3504297/8071176/375-1.pdf, doi:10.2307/3504297.

[15] W. A. Laurie, E. M. Lang, and C. P. Groves. Rhinoceros unicornis. *Mammalian Species*, (211):1–6, 12 1983. arXiv:https://academic.oup.com/mspecies/article-pdf/doi/10.2307/3504002/8070033/211-1.pdf, doi:10.2307/3504002.

[16] Dana N. Lee, Richard W. Dolman, and Jr. Leslie, David M. Oryx callotis (Artiodactyla: Bovidae). *Mammalian Species*, 45(897):1–11, 03 2013. arXiv:https://academic.oup.com/mspecies/article-pdf/45/897/1/8269181/45-897-1.pdf, doi:10.1644/897.1.

[17] Jr. Leslie, David M. Boselaphus Tragocamelus (Artiodactyla: Bovidae). *Mammalian Species*, (813):1–16, 06 2008. arXiv:https://academic.oup.com/mspecies/article-pdf/doi/10.1644/813.1/24460934/813.pdf, doi:10.1644/813.1.

[18] Jr. Leslie, David M. and Koustubh Sharma. Tetracerus quadricornis (Artiodactyla: Bovidae). *Mammalian Species*, (843):1–11, 09 2009. arXiv:https://academic.oup.com/mspecies/article-pdf/doi/10.1644/843.1/24461167/843.pdf, doi:10.1644/843.1.

[19] Viktor A. Makarov and Aleksandra A. Panyutina. Running of the feathertail glider (Acrobates pygmaeus) on level ground: Gaits. *Journal of Experimental Zoology Part A: Ecological and Integrative Physiology*, 337(4):366–380, 2022. doi:10.1002/jez.2573.

[20] Vratislav Mazák. Panthera tigris. *Mammalian Species*, (152):1–8, 05 1981. arXiv:https://academic.oup.com/mspecies/article-pdf/doi/10.2307/3504004/8070903/152-1.pdf, doi:10.2307/3504004.

[21] Mary Meagher. Bison bison. *Mammalian Species*, (266):1–8, 06 1986. arXiv:https://academic.oup.com/mspecies/article-pdf/doi/10.1093/mspecies/266.1/8071005/266-1.pdf, doi:10.1093/mspecies/266.1.

[22] L. David Mech. Canis lupus. *Mammalian Species*, (37):1–6, 05 1974. arXiv:https://academic.oup.com/mspecies/article-pdf/doi/10.2307/3503924/8071170/37-1.pdf, doi:10.2307/3503924.

[23] Bart W. O'Gara. Antilocapra americana. *Mammalian Species*, (90):1–1, 01 1978. arXiv:https://academic.oup.com/mspecies/article-pdf/doi/10.2307/3504049/8071855/90-1.pdf, doi:10.2307/3504049.

[24] Lindsay A. Pappas. Taurotragus oryx. *Mammalian Species*, (689):1–5, 07 2002. arXiv:https://academic.oup.com/mspecies/article-pdf/doi/10.1644/0.689.1/8070663/689-1.pdf, doi:10.1644/0.689.1.

[25] Amanda M Veals, Alexandra D Burnett, Marina Morandini, Marine Drouilly, and John L Koprowski. Caracal caracal (Carnivora: Felidae). *Mammalian Species*, 52(993):71–85, 12 2020. arXiv:https://academic.oup.com/mspecies/article-pdf/52/993/71/34667545/seaa006.pdf, doi:10.1093/mspecies/seaa006.

[26] I Mauricio Vela-Vargas, Jeffrey P Jorgenson, José F González-Maya, and John L Koprowski. Tremarctos ornatus (Carnivora: Ursidae). *Mammalian Species*, 53(1006):78–94, 07 2021. arXiv:https://academic.oup.com/mspecies/article-pdf/53/1006/78/39039172/seab008.pdf, doi:10.1093/mspecies/seab008.

[27] Xiaoming Wang and Robert S. Hoffmann. Pseudois nayaur and Pseudois schaeferi. *Mammalian Species*, (278):1–6, 02 1987. arXiv:https://academic.oup.com/mspecies/article-pdf/doi/10.2307/3503993/8071029/278-1.pdf, doi:10.2307/3503993.

[28] Yoram Yom-Tov, Heinrich Mendelssohn, and Colin P. Groves. Gazella dorcas. *Mammalian Species*, (491):1–6, 06 1995. arXiv:https://academic.oup.com/mspecies/article-pdf/doi/10.2307/3504254/8071365/491-1.pdf, doi:10.2307/3504254.

Non-peer reviewed

[29] Japanese serow, 2023. URL: https://en.wikipedia.org/wiki/Japanese_serow.

[30] Species: Didelphis virginiana, 2023. URL: https://www.gbif.org/species/2439923, doi:10.15468/39omei.

[31] Species: Genetta genetta, 2023. URL: https://www.gbif.org/species/5219362, doi:10.15468/39omei.

[32] Species: Helarctos malayanus, 2023. URL: https://www.gbif.org/species/2433403, doi:10.15468/39omei.

[33] Species: Hemitragus jemlahicus, 2023. URL: https://www.gbif.org/species/5220174, doi:10.15468/39omei.

[34] Species: Hippotragus equinus, 2023. URL: https://www.gbif.org/species/2441030, doi:10.15468/39omei.

[35] Species: Kobus ellipsiprymnus, 2023. URL: https://www.gbif.org/species/5220160, doi:10.15468/39omei.

[36] Species: Lama glama, 2023. URL: https://www.gbif.org/species/5220190, doi:10.15468/39omei.

[37] Species: Lasiorhinus latifrons, 2023. URL: https://www.gbif.org/species/5219996, doi:10.15468/39omei.

[38] Species: Leopardus pardalis, 2023. URL: https://www.gbif.org/species/2434982, doi:10.15468/39omei.

[39] Species: Lynx rufus, 2023. URL: https://www.gbif.org/species/2435246, doi:10.15468/39omei.

[40] Species: Oreamnos americanus, 2023. URL: https://www.gbif.org/species/2441151, doi:10.15468/39omei.

[41] Species: Oryx gazella, 2023. URL: https://www.gbif.org/species/5220166, doi:10.15468/39omei.

[42] Species: Ptilocercus lowii, 2023. URL: https://www.gbif.org/species/2436257, doi:10.15468/39omei.

[43] Species: Redunca arundinum, 2023. URL: https://www.gbif.org/species/2441037, doi:10.15468/39omei.

[44] Species: Tolypeutes matacus, 2023. URL: https://www.gbif.org/species/2440792, doi:10.15468/39omei.

[45] Species: Tragelaphus scriptus, 2023. URL: https://www.gbif.org/species/5220182, doi:10.15468/39omei.

[46] Species: Tupaia minor, 2023. URL: https://www.gbif.org/species/2436283, doi:10.15468/39omei.

[47] Species: Tupaia tana, 2023. URL: https://www.gbif.org/species/2436303, doi:10.15468/39omei.

[48] Species: Vombatus ursinus, 2023. URL: https://www.gbif.org/species/2440301, doi:10.15468/39omei.

[49] wolverinefoundation, 2023. URL: https://wolverinefoundation.org/?guid=on.

[50] R. Anderson. Castor canadensis, 2002. (On-line), Animal Diversity Web. URL: https://animaldiversity.org/accounts/Castor_canadensis/.

[51] N Anna Toenjes. Felis catus, 2014. (On-line), Animal Diversity Web. URL: https://animaldiversity.org/accounts/Felis_catus/.

[52] A. Auman, Fye R., and Dewey T. Eudorcas thomsonii, 2009. (On-line), Animal Diversity Web. URL: https://animaldiversity.org/accounts/Eudorcas_thomsonii/.

[53] M. Bador. Tupaia palawanensis, 2019. (On-line), Animal Diversity Web. URL: https://animaldiversity.org/accounts/Tupaia_palawanensis/.

[54] J. Barnard. Antidorcas marsupialis, 1999. (On-line), Animal Diversity Web. URL: https://animaldiversity.org/accounts/Antidorcas_marsupialis/.

[55] L. Bies. Melursus ursinus, 2002. (On-line), Animal Diversity Web. URL: https://animaldiversity.org/accounts/Melursus_ursinus/.

[56] J. Black. Dasyurus hallucatus, 2013. (On-line), Animal Diversity Web. URL: https://animaldiversity.org/accounts/Dasyurus_hallucatus/.

[57] A. Bunker. Taxidea taxus, 2007. (On-line), Animal Diversity Web. URL: https://animaldiversity.org/accounts/Canis_simensis/.

[58] T. Canniff. Leptailurus serval, 2011. (On-line), Animal Diversity Web. URL: https://animaldiversity.org/accounts/Leptailurus_serval/.

[59] E. Cohen. Naemorhedus goral, 2009. (On-line), Animal Diversity Web. URL: https://animaldiversity.org/accounts/Naemorhedus_goral/.

[60] G. D'Elia. Myocastor coypus, 1999. (On-line), Animal Diversity Web. URL: https://animaldiversity.org/accounts/Myocastor_coypus/.

[61] T. Dewey. Odocoileus virginianus, 2003. (On-line), Animal Diversity Web. URL: https://animaldiversity.org/accounts/Odocoileus_virginianus/.

[62] K. DeWitt. Cephalophus silvicultor, 2006. (On-line), Animal Diversity Web. URL: https://animaldiversity.org/accounts/Cephalophus_silvicultor/.

[63] D. Dulal. Ceratotherium simum, 2017. (On-line), Animal Diversity Web. URL: https://animaldiversity.org/accounts/Ceratotherium_simum/.

[64] S. Elder. Ovibos moschatus, 2015. (On-line), Animal Diversity Web. URL: https://animaldiversity.org/accounts/Ovibos_moschatus/.

[65] M. Ewacha. Oreotragus oreotragus, 2013. (On-line), Animal Diversity Web. URL: https://animaldiversity.org/accounts/Oreotragus_oreotragus/.

[66] B. Fahey and A. Kinder. Sarcophilus harrisii, 2001. (On-line), Animal Diversity Web. URL: https://animaldiversity.org/accounts/Sarcophilus_harrisii/.

[67] M. Hart. Echymipera rufescens, 2012. (On-line), Animal Diversity Web. URL: https://animaldiversity.org/accounts/Echymipera_rufescens/.

[68] K. Holmes. Neofelis nebulosa, 2009. (On-line), Animal Diversity Web. URL: https://animaldiversity.org/accounts/Neofelis_nebulosa/.

[69] J. Humfleet. Hemibelideus lemuroides, 2006. (On-line), Animal Diversity Web. URL: https://animaldiversity.org/accounts/Hemibelideus_lemuroides/.

[70] N. Karkala. Elephas maximus, 2016. (On-line), Animal Diversity Web. URL: https://animaldiversity.org/accounts/Elephas_maximus/.

[71] S. Maisano. Giraffa camelopardalis, 2006. (On-line), Animal Diversity Web. URL: https://animaldiversity.org/accounts/Giraffa_camelopardalis/.

[72] M. McPhee. Hystrix cristata, 2003. (On-line), Animal Diversity Web. URL: https://animaldiversity.org/accounts/Hystrix_cristata/.

[73] A. Mileski. Capra hircus, 2004. (On-line), Animal Diversity Web. URL: https://animaldiversity.org/accounts/Capra_hircus/.

[74] D. Miller. Bubalus depressicornis, 2002. (On-line), Animal Diversity Web. URL: https://animaldiversity.org/accounts/Bubalus_depressicornis/.

[75] M. Misuraca. Odocoileus hemionus, 1999. (On-line), Animal Diversity Web. URL: https://animaldiversity.org/accounts/Odocoileus_hemionus/.

[76] J. Ng. Bos taurus, 2001. (On-line), Animal Diversity Web. URL: https://animaldiversity.org/accounts/Bos_taurus/.

[77] W. Nocon. Antilope cervicapra, 1999. (On-line), Animal Diversity Web. URL: https://animaldiversity.org/accounts/Antilope_cervicapra/.

[78] M. Oliphant. Bos grunniens, 2003. (On-line), Animal Diversity Web. URL: https://animaldiversity.org/accounts/Bos_grunniens/.

[79] J. Pietryga. Urogale everetti, 2002. (On-line), Animal Diversity Web. URL: https://animaldiversity.org/accounts/Urogale_everetti/.

[80] E. Raffo. Geocapromys brownii, 2000. (On-line), Animal Diversity Web. URL: https://animaldiversity.org/accounts/Geocapromys_brownii/.

[81] E. Ratzloff. Orycteropus afer, 2011. (On-line), Animal Diversity Web. URL: https://animaldiversity.org/accounts/Orycteropus_afer/.

[82] J. Saari. Bos javanicus, 2002. (On-line), Animal Diversity Web. URL: https://animaldiversity.org/accounts/Bos_javanicus/.

[83] N. Shefferly. Taxidea taxus, 1999. (On-line), Animal Diversity Web. URL: https://animaldiversity.org/accounts/Taxidea_taxus/.

[84] L. Siciliano Martina. Caluromysiops irrupta, 2014. (On-line), Animal Diversity Web. URL: https://animaldiversity.org/accounts/Caluromysiops_irrupta/.

[85] L. Siciliano Martina. Didelphis marsupialis, 2014. (On-line), Animal Diversity Web. URL: https://animaldiversity.org/accounts/Didelphis_marsupialis/.

[86] N. Succop. Dicerorhinus sumatrensi, 2023. (On-line), Animal Diversity Web. URL: https://animaldiversity.org/accounts/Dicerorhinus_sumatrensis/.

[87] S. Verjinski. Dasyurus maculatus, 2013. (On-line), Animal Diversity Web. URL: https://animaldiversity.org/accounts/Dasyurus_maculatus/.

[88] C. Weber. Erethizon dorsatum, 2004. (On-line), Animal Diversity Web. URL: https://animaldiversity.org/accounts/Erethizon_dorsatum/.

[89] J. Wells. Tapirus bairdii, 2009. (On-line), Animal Diversity Web. URL: https://animaldiversity.org/accounts/Tapirus_bairdii/.

Percentage of the stride period

During galloping, the elbow undergoes two instances of extension: the first, at the end of the swing phase (after-swing), while the second, during the mid-stance phase [12, 14, 40] (fig. S1). To determine the duration of these instances, we referred to data provided by Tokuriki [40] who reported the angular movement of the elbow for both forelimbs (i.e., the leading and trailing forelimbs) for one gait cycle of dogs. We used the PlotDigitizer tool to extract relevant data from plots in his article. We calculated the average of 10 measurements to evaluate the extension periods as a percentage of the total gait cycle.

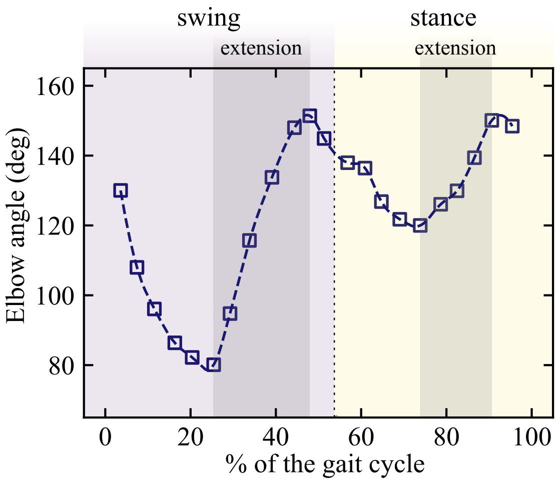

Figure S1: Angular movements of the elbow during galloping for the trailing limb. Data were taken from Tokuriki [40] with the PlotDigitizer tool [1].

We analyzed the rate of change of the elbow angle during each extension period to determine the fastest period of extension. We found that the extension period in the swing phase was the fastest for both the leading and trailing forelimbs. We calculated the average percentage of the fastest extension for both forelimbs, which was 25.4% of the entire gait cycle.

Moment of inertia of the lower forearm about the elbow rotation axis

Coatham et al. [8] used convex hulls to estimate body segment parameters (BSPs) in various extant mammals. They used computed tomography (CT) scans to generate virtual segments of both the skeleton and skin. They then developed regression equations to link BSPs obtained from convex hulls reconstructed from the bones with the skin derived from the CT scan.

We used the 3D digital files of reconstructed body segments of quadrupedal mammals from Coatham et al. [8], available in their supplementary data [9]. Using these files, we calculated the mass, center of mass (CoM), moment of inertia (MoI) at the CoM, and the location of the CoM within the forearm ($d_{forearm}$), arm (d_{arm}), and hand (d_{hand}) (fig. S2). A nominal density of 1000 Kg m^{-3} was assumed [8]. Only the left side of quadrupedal mammals was considered (or the right when the left was unavailable); primates and knuckle-walking mammals were excluded.

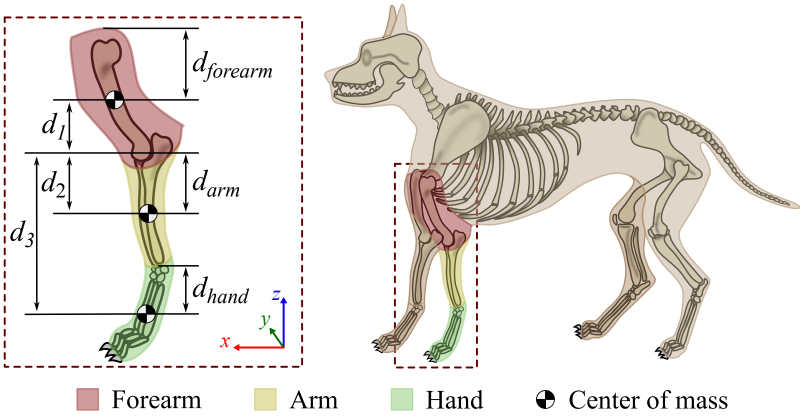

Figure S2: Segments of the animals provided by Coatham et al. [8] and distances used to calculate the moment of inertia about the elbow of each segment. $d_{forearm}$: location of the forearm center of mass, d_{arm}: location of the arm center of mass, d_{hand}: location of the hand center of mass, d_1: distance between the center of mass of the forearm and elbow. d_2: distance between the center of mass of the arm and elbow. d_3: distance between the center of mass of the hand and elbow.

The Huygens-Steiner theorem, also known as the parallel axes theorem[1], was used to determine the moment of inertia (MoI) of each body segment around the elbow eq. (1):

$$I_{elbow_{segment}} = I_{0_{segment}} + m_{segment} d_i^2 \tag{1}$$

where, $I_{elbow_{segment}}$ is the MoI of each segment (forearm, arm, hand) around the elbow, $I_{0_{segment}}$ the MoI of each segment around its center of mass, $m_{segment}$ the mass of each segment, and d_i the perpendicular distance between the center of mass of each segment and the elbow. The values of d_i for the forearm, arm, and hand were calculated using eq. (2):

$$\begin{aligned} d_1 &= l - d_{forearm} \\ d_2 &= d_{arm} \\ d_3 &= l_r + d_{hand} \end{aligned} \tag{2}$$

where l, and l_r were assumed as the length of the humerus and radius, respectively, and determined using allometric relationships. The mass of each animal was taken from various sources in the literature (table S3). See table S3 for the summary of the MoI of the segments and the lower arm ($I_{elbow_{hand}} + I_{elbow_{forearm}}$) at the elbow.

[1]This theorem enables the calculation of the MoI about any axis (I) using the mass (m), the MoI about a parallel axis passing through the center of gravity (I_0), and the perpendicular distance between the axes (d) through the equation $I = I_0 + md^2$.

Table S3: Forelimb segments properties.

**Species**	**mass**	**Ref**	$m_{forearm}$	m_{hand}	m_{arm}	$d_{forearm}$	d_{hand}	d_{arm}	$I_{0_{forearm}}$	$I_{0_{hand}}$	$I_{0_{arm}}$	l [i]	l_r [ii]	$I_{elbow_{forearm}}$ [iii]	$I_{elbow_{hand}}$ [iii]	$I_{elbow_{arm}}$ [iii]	$I_{elbow_{forelimb}}$ [iv]	$I_{elbow_{lowerarm}}$ [v]
Acinonyx jubatus	39.00	[7]	1.31E+00	2.07E-01	4.60E-01	1.17E-01	7.75E-02	1.37E-01	5.46E-03	4.51E-04	2.56E-03	1.56E-01	1.90E-01	7.47E-03	1.52E-02	1.11E-02	3.38E-02	2.64E-02
Cynomys ludovicianus	1.13	[10]	1.70E-02	2.17E-03	9.55E-03	1.84E-02	9.93E-03	2.80E-02	2.53E-06	1.00E-07	1.37E-06	5.20E-02	4.49E-02	2.17E-05	6.63E-06	8.85E-06	3.71E-05	1.55E-05
Felis catus	4.75	[4]	5.36E-02	1.07E-02	2.96E-02	5.54E-02	5.36E-02	4.93E-02	3.31E-05	2.41E-06	1.91E-05	8.12E-02	8.07E-02	6.88E-05	1.96E-04	9.11E-05	3.56E-04	2.87E-04
Hemicentetes semispinosus	0.18	[23]	5.12E-03	5.95E-04	1.84E-03	2.23E-02	4.20E-03	1.67E-02	4.40E-07	2.00E-08	1.10E-07	2.95E-02	2.13E-02	7.00E-07	4.07E-07	6.23E-07	1.73E-06	1.03E-06
Hydrochoeris hydrochaeris	50.50	[32]	5.19E-01	2.00E-01	3.57E-01	8.30E-02	5.33E-02	7.31E-02	1.32E-03	2.28E-04	7.31E-04	1.69E-01	2.11E-01	5.16E-03	1.42E-02	2.64E-03	2.20E-02	1.68E-02
Leopardus geoffroyi	4.30	[22]	7.33E-02	1.39E-02	4.15E-02	5.55E-02	2.77E-02	4.79E-02	4.70E-05	3.67E-06	2.44E-05	7.88E-02	7.75E-02	8.68E-05	1.58E-04	1.20E-04	3.64E-04	2.78E-04
Lutra lutra	6.70	[10]	1.10E-01	1.99E-02	8.35E-02	5.17E-02	3.01E-02	6.52E-02	6.86E-05	5.81E-06	5.62E-05	9.04E-02	9.28E-02	2.34E-04	3.06E-04	4.11E-04	9.51E-04	7.16E-04
Martes melampus	1.25	[2]	4.11E-02	9.44E-03	1.95E-02	3.59E-02	2.28E-02	3.76E-02	1.48E-05	1.98E-06	5.71E-06	5.37E-02	4.69E-02	2.79E-05	4.79E-05	3.32E-05	1.09E-04	8.11E-05
Meles anakuma	7.45	[36]	2.03E-01	2.98E-02	9.75E-02	6.31E-02	2.47E-02	5.75E-02	1.97E-04	8.16E-06	6.60E-05	9.34E-02	9.68E-02	3.84E-04	4.48E-04	3.88E-04	1.22E-03	8.36E-04
Nyctereutes procyonoides	5.23	[17]	8.04E-02	2.05E-02	3.71E-02	6.18E-02	2.64E-02	4.07E-02	7.04E-05	5.80E-06	2.08E-05	8.37E-02	8.39E-02	1.09E-04	2.56E-04	8.24E-05	4.47E-04	3.38E-04
Paguma larvata	4.30	[28]	4.35E-02	1.18E-02	2.84E-02	3.75E-02	2.16E-02	3.61E-02	2.14E-05	2.13E-06	1.06E-05	7.88E-02	7.75E-02	9.57E-05	1.18E-04	4.77E-05	2.62E-04	1.66E-04
Procyon lotor	7.50	[27]	1.48E-01	2.24E-02	6.75E-02	5.23E-02	3.00E-02	4.30E-02	1.41E-04	6.96E-06	5.88E-05	9.36E-02	9.71E-02	3.94E-04	3.69E-04	1.84E-04	9.47E-04	5.53E-04
Rattus norvegicus	0.32	[5]	6.27E-03	6.67E-04	2.64E-03	1.91E-02	8.33E-03	1.64E-02	6.80E-07	2.00E-08	2.10E-07	3.52E-02	2.69E-02	2.31E-06	8.49E-07	9.19E-07	4.08E-06	1.77E-06
Sus scrofa	169.00	[45]	1.28E-01	5.16E-02	8.96E-02	5.53E-02	3.99E-05	4.73E-02	1.23E-04	3.99E-05	8.71E-05	2.46E-01	3.45E-01	4.78E-03	6.17E-03	2.87E-04	1.12E-02	6.46E-03
Ursus maritimus	265.00	[19]	2.56E+00	6.61E-01	2.29E+00	1.07E-01	7.19E-02	1.27E-01	2.09E-02	1.88E-03	1.71E-02	2.83E-01	4.14E-01	1.00E-01	1.58E-01	5.43E-02	3.12E-01	2.12E-01

The masses are in kilograms (kg), the distances in meters (m), and the moments of inertia in kg m^2.

[i] Calculated with the allometric relationship found in this study.

[ii] Calculated with the allometric relationship reported by Christiansen [6].

[iii] Calculated with the theorem of parallel axes.

[iv] The addition of the MoI about the elbow of the three segments of the forelimb.

[v] The addition of the MoI about the elbow of the arm and hand.

We established power-law relationships between the MoI of the lower arm around the elbow rotation axis ($I_{elbow_{lowerarm}}$) to body mass (M): $Y = aM^b$, where Y is the $I_{elbow_{lowerarm}}$. The variables were $\log_{10}$-transformed prior analysis. For the regression, we used the standardized major axis (SMA) line-fitting method with the 'smatr' package for R [35, 44]. We got that $I_{elbow_{lowerarm}} \propto M^{1.78}$ (fig. S3).

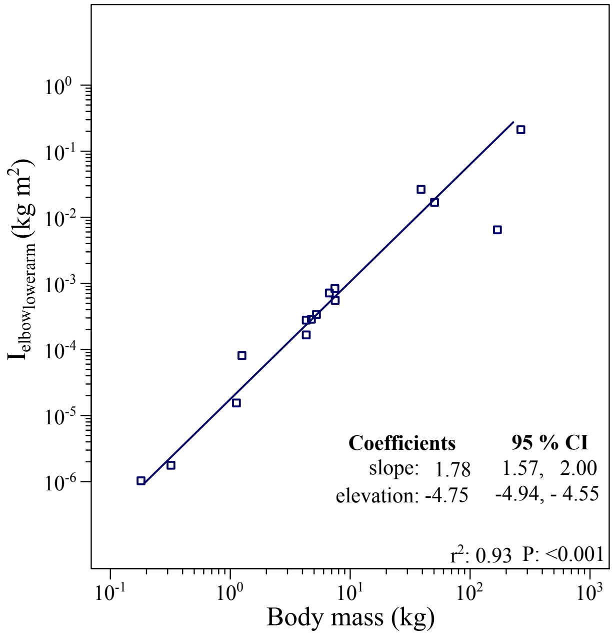

Figure S3: Allometry for the MoI regarding the elbow of the lower arm. We tested the null hypothesis 'H_0: variables are uncorrelated'.

Regressions

Phylogenetic generalized least squares models

The histograms of the elevations, slopes, and λ obtained for the 10 000 trees are shown in fig. S4. The average value for each of these parameters is marked by a green dashed line. In the table S4 are reported the results of the PGLS of the tree closest with values closest to the average (tree 1146, see [29] to access the 10 000 trees in nexus file format)[2].

[2]available at https://doi.org/10.5281/zenodo.7993776

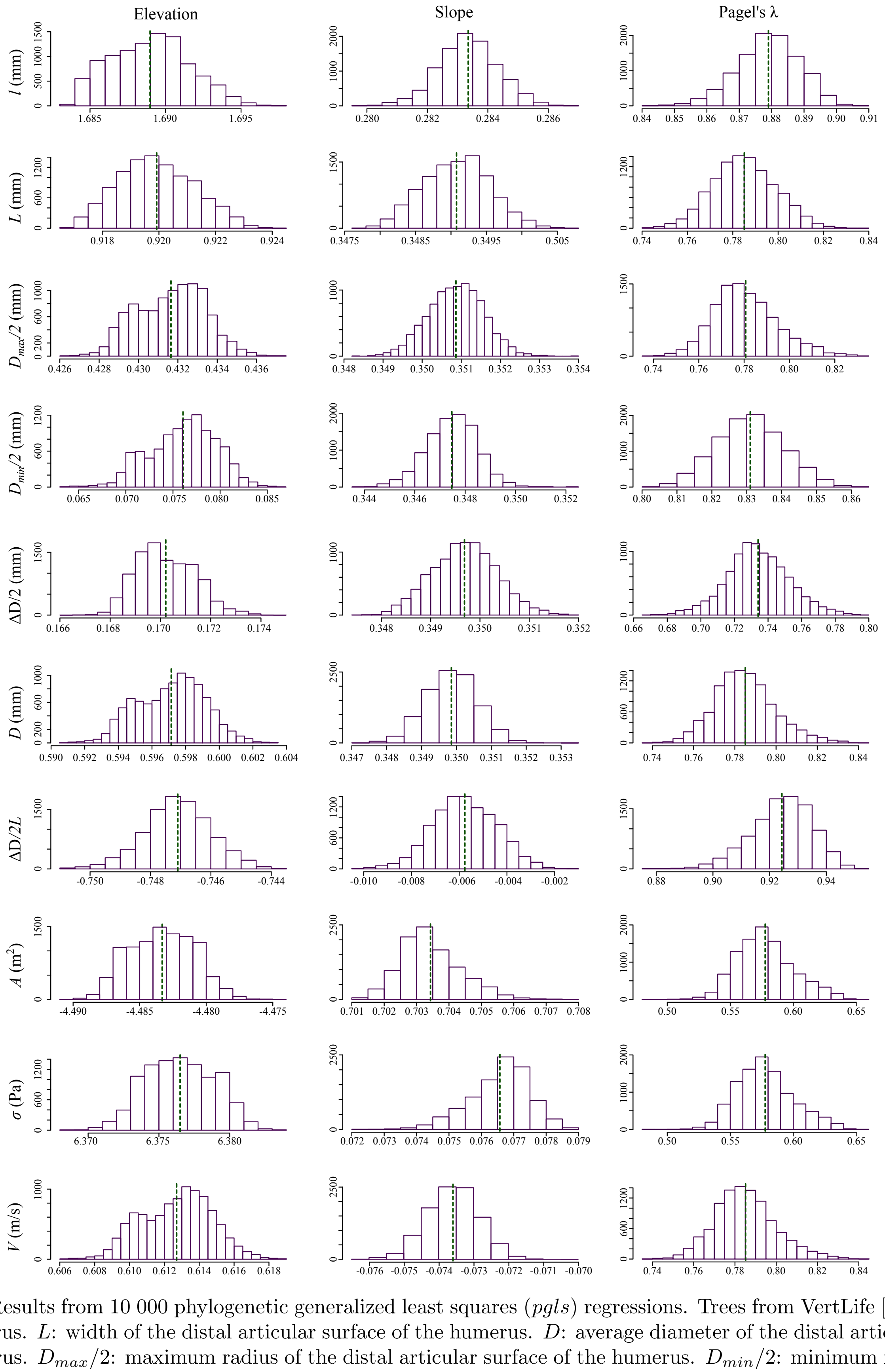

Figure S4: Results from 10 000 phylogenetic generalized least squares ($pgls$) regressions. Trees from VertLife [41].l: length of the humerus. L: width of the distal articular surface of the humerus. D: average diameter of the distal articular surface of the humerus. $D_{max}/2$: maximum radius of the distal articular surface of the humerus. $D_{min}/2$: minimum radius of the distal articular surface of the humerus. ΔR: arithmetic difference between $D_{max}/2$ and $D_{min}/2$. A: projected area of the distal articular surface of the humerus ($D \times L$). σ: average contact stress at the distal articular surface of the humerus. V: sliding speed at the distal articular surface of the humerus.

Table S4: Phylogenetic least squares (PGLS) regression elevations and slopes.

Predictor: Body mass M (kg)

Response		Estimate	95 % CI	
l (mm)	slope	0.28	0.26	0.30
	elev.	1.69	1.57	1.81
	λ	0.87	0.67	0.95
	r^2: 0.89		$P < .001$	
L (mm)	slope	0.35	0.33	0.37
	elev.	0.92	0.81	1.03
	λ	0.79	0.35	0.91
	r^2: 0.94		$P < .001$	
D (mm)	slope	0.35	0.33	0.37
	elev.	0.60	0.49	0.70
	λ	0.78	0.39	0.90
	r^2: 0.94		$P < .001$	
$D_{max}/2$ (mm)	slope	0.35	0.33	0.37
	elev.	0.43	0.32	0.54
	λ	0.77	0.45	0.92
	r^2: 0.94		$P < .001$	
$D_{min}/2$ (mm)	slope	0.35	0.32	0.37
	elev.	0.07	-0.09	0.24
	λ	0.83	0.61	0.94
	r^2: 0.89		$P < .001$	
$\Delta D/2$ (mm)	slope	0.35	0.33	0.37
	elev.	0.17	0.04	0.30
	λ	0.71	0.44	0.89
	r^2: 0.89		$P < .001$	
$\frac{\Delta D}{2L}$	slope	0.01	-0.03	0.02
	elev.	-0.75	-0.94	-0.56
	λ	0.93	0.74	0.98
	r^2: 0.002		$P = .638$	
A (m^2)	slope	0.70	0.68	0.73
	elev.	-4.48	-4.62	-4.35
	λ	0.58	0.13	0.86
	r^2: 0.96		$P < .001$	
σ (Pa)	slope	0.08	0.05	0.10
	elev.	6.38	6.24	6.51
	λ	0.58	0.13	0.86
	r^2: 0.22		$P < .001$	
V $\frac{m}{s}$	slope	-0.07	-0.09	-0.06
	elev.	0.61	0.51	0.72
	λ	0.78	0.44	0.93
	r^2: 0.41		$P < .001$	

Predictor: l(mm)

Response		Estimate	95 % CI	
L (mm)	slope	1.17	1.10	1.23
	elev.	-1.03	-1.26	-0.81
	λ	0.95	0.86	0.98
	r^2: 0.91		$P < .001$	
D (mm)	slope	1.17	1.10	1.23
	elev.	-1.37	-1.54	-1.20
	λ	0.93	0.83	0.97
	r^2: 0.94		$P < .001$	
$D_{max}/2$ (mm)	slope	1.18	1.12	1.23
	elev.	-1.54	-1.71	-1.38
	λ	0.89	0.75	0.96
	r^2: 0.94		$P < .001$	
$D_{min}/2$ (mm)	slope	1.17	1.09	1.25
	elev.	-1.89	-2.12	-1.65
	λ	0.90	0.77	0.96
	r^2: 0.89		$P < .001$	
$\Delta D/2$ (mm)	slope	1.17	1.10	1.24
	elev.	-1.79	-1.98	-1.61
	λ	0.76	0.54	0.90
	r^2: 0.90		$P < .001$	
$\frac{\Delta D}{2L}$	slope	0.00	-0.08	0.08
	elev.	-0.76	-1.00	-0.52
	λ	0.92	0.72	0.98
	r^2: 3.6e-5		$P = .950$	
A (m^2)	slope	2.34	2.22	2.45
	elev.	-8.40	-8.75	-8.05
	λ	0.93	0.82	0.97
	r^2: 0.94		$P < .001$	
σ (Pa)	slope	0.15	0.06	0.25
	elev.	6.14	5.91	6.37
	λ	0.53	0.06	0.85
	r^2: 0.08		$P = .002$	
V $\frac{m}{s}$	slope	-0.17	-0.23	-0.11
	elev.	0.88	0.72	1.04
	λ	0.72	0.24	0.91
	r^2: 0.21		$P < .001$	

Predictor: L (mm)

Response		Estimate	95 % CI	
D (mm)	slope	0.96	0.92	1.00
	elev.	-0.29	-0.45	-0.12
	λ	0.99	0.95	1.00
	r^2: 0.95		$P < .001$	
$D_{max}/2$ (mm)	slope	0.98	0.94	1.02
	elev.	-0.47	-0.62	-0.31
	λ	0.97	0.92	0.99
	r^2: 0.95		$P < .001$	
$D_{min}/2$ (mm)	slope	0.98	0.92	1.04
	elev.	-1.89	-2.14	-1.65
	λ	0.93	0.83	0.97
	r^2: 0.89		$P < .001$	
$\Delta D/2$ (mm)	slope	0.96	0.90	1.03
	elev.	-0.71	-0.78	-0.65
	λ	0.93	0.76	0.98
	r^2: 0.89		$P < .001$	
$\frac{\Delta D}{2L}$	slope	-0.03	-0.10	0.03
	elev.	-0.71	-0.91	-0.51
	λ	0.93	0.76	0.98
	r^2: 0.01		$P = .287$	
A (m^2)	slope	1.96	1.92	2.00
	elev.	-6.29	-6.45	-6.12
	λ	0.99	0.95	1.00
	r^2: 0.99		$P < .001$	
σ (Pa)	slope	0.13	0.04	0.21
	elev.	6.28	6.12	6.44
	λ	0.50	NA	0.85
	r^2: 0.08		$P = .002$	
V $\frac{m}{s}$	slope	-0.16	-0.20	-0.11
	elev.	0.75	0.64	0.85
	λ	0.64	0.13	0.89
	r^2: 0.27		$P < .001$	

Predictor: D (mm)

Response		Estimate	95 % CI	
$D_{max}/2$ (mm)	slope	1.00	0.99	1.02
	elev.	-0.17	-0.21	-0.13
	λ	0.86	0.64	0.96
	r^2: 0.99		$P < .001$	
$D_{min}/2$ (mm)	slope	1.00	0.97	1.04
	elev.	-0.83	-1.03	-0.62
	λ	0.76	0.51	0.91
	r^2: 0.96		$P < .001$	
$\Delta D/2$ (mm)	slope	0.99	0.93	1.04
	elev.	-0.42	-0.55	-0.28
	λ	0.82	0.59	0.94
	r^2: 0.92		$P < .001$	
$\frac{\Delta D}{2L}$	slope	0.00	-0.07	0.06
	elev.	-0.75	-0.94	-0.56
	λ	0.92	0.72	0.98
	r^2: 9.9e-5		$P = .918$	
A (m^2)	slope	1.99	1.95	2.03
	elev.	-5.66	-5.82	-5.50
	λ	0.98	0.94	1.00
	r^2: 0.99		$P < .001$	
σ (Pa)	slope	0.13	0.05	0.21
	elev.	6.32	6.18	6.46
	λ	0.47	NA	0.84
	r^2: 0.09		$P = .002$	
V $\frac{m}{s}$	slope	-0.14	-0.19	-0.09
	elev.	0.68	0.56	0.80
	λ	0.73	0.19	0.91
	r^2: 0.21		$P < .001$	

Predictor: $D_{max}/2$ (mm)

Response		Estimate	95 % CI	
$D_{min}/2$ (mm)	slope	1.00	0.95	1.04
	elev.	-0.36	-0.46	-0.25
	λ	0.82	0.61	0.93
	r^2: 0.95		$P < .001$	
$\Delta D/2$ (mm)	slope	1.00	0.95	1.04
	elev.	-0.26	-0.36	-0.16
	λ	0.77	0.52	0.92
	r^2: 0.95		$P < .001$	
$\frac{\Delta D}{2L}$	slope	0.01	-0.05	0.08
	elev.	-0.76	-0.96	-0.56
	λ	0.93	0.75	0.98
	r^2: 1.5e-3		$P = .686$	
A (m^2)	slope	1.96	1.91	2.01
	elev.	-5.32	-5.48	-5.16
	λ	0.94	0.86	0.98
	r^2: 0.98		$P < .001$	
σ (Pa)	slope	0.13	0.05	0.21
	elev.	6.34	6.21	6.48
	λ	0.49	NA	0.85
	r^2: 0.09		$P = .002$	
V $\frac{m}{s}$	slope	-0.14	-0.19	-0.09
	elev.	0.66	0.54	0.77
	λ	0.73	0.20	0.91
	r^2: 0.22		$P < .001$	

Predictor: $D_{min}/2$ (mm)

Response		Estimate	95 % CI	
$\Delta D/2$ (mm)	slope	0.89	0.81	0.98
	elev.	0.13	-0.08	0.34
	λ	0.84	0.60	0.95
	r^2: 0.80		$P < .001$	
$\frac{\Delta D}{2L}$	slope	-0.04	-0.10	0.02
	elev.	-0.74	-0.94	-0.53
	λ	0.94	0.79	0.98
	r^2: 0.02		$P = .157$	
A (m^2)	slope	1.86	1.77	1.95
	elev.	-4.58	-4.86	-4.31
	λ	0.91	0.75	0.96
	r^2: 0.94		$P < .001$	
σ (Pa)	slope	0.12	0.04	0.19
	elev.	6.39	6.27	6.52
	λ	0.47	NA	0.85
	r^2: 0.08		$P = .004$	
V $\frac{m}{s}$	slope	-0.12	-0.18	-0.07
	elev.	0.60	0.48	0.72
	λ	0.77	0.25	0.93
	r^2: 0.16		$P < .001$	

Predictor: ΔR (mm)

Response		Estimate	95 % CI	
$\frac{\Delta D}{2L}$	slope	0.08	0.01	0.14
	elev.	-0.78	-0.96	-0.60
	λ	0.93	0.71	0.98
	r^2: 0.05		$P = .015$	
A (m^2)	slope	1.86	1.76	1.96
	elev.	0.81	0.44	1.18
	λ	0.84	0.59	0.96
	r^2: 0.92		$P < .001$	
σ (Pa)	slope	0.13	0.05	0.20
	elev.	6.76	6.52	6.99
	λ	0.50	0.03	0.84
	r^2: 0.09		$P = .002$	
V $\frac{m}{s}$	slope	-0.14	-0.19	-0.09
	elev.	0.20	0.04	0.36
	λ	0.68	0.14	0.90
	r^2: 0.21		$P < .001$	

Predictor: $\Delta R/L$

Response		Estimate	95 % CI	
A (m^2)	slope	-0.29	-1.20	0.63
	elev.	-4.15	-5.90	-2.40
	λ	1.00	0.98	NA
	r^2: 3.5e-3		$P = .538$	
σ (Pa)	slope	0.10	-0.19	0.40
	elev.	6.48	6.25	6.71
	λ	0.72	0.22	0.91
	r^2: 0.004		$P = .495$	
V $\frac{m}{s}$	slope	0.11	-0.08	0.30
	elev.	0.61	0.42	0.81
	λ	0.89	0.70	0.96
	r^2: 0.01		$P = .241$	

Predictor: A(m^2)

Response		Estimate	95 % CI	
σ (Pa)	slope	0.06	0.02	0.10
	elev.	6.68	6.48	6.89
	λ	0.48	NA	0.85
	r^2: 0.08		$P = .002$	
V $\frac{m}{s}$	slope	-0.07	-0.10	-0.05
	elev.	0.26	0.12	0.41
	λ	0.69	0.16	0.90
	r^2: 0.24		$P < .001$	

Predictor: σ (Pa)

Response		Estimate	95 % CI	
V $\frac{m}{s}$	slope	-0.56	-0.60	-0.52
	elev.	4.15	3.87	4.44
	λ	0.99	0.94	NA
	r^2: 0.87		$P < .001$	

- l: the length of the humerus.
- L: the width of the distal articular surface of the humerus.
- D: average diameter of the distal articular surface of the humerus.
- $D_{max}/2$: maximum radius of the distal articular surface of the humerus.
- $D_{min}/2$: minimum radius of the distal articular surface of the humerus.
- $\Delta D/2$: arithmetic difference between $D_{max}/2$ and $D_{min}/2$.
- A: projected area of the distal articular surface of the humerus calculated as $D \times L$.
- σ: average contact stress at the distal articular surface of the humerus.
- V: sliding speed at the distal articular surface of the humerus.
- Columns colors are not related to the values of the data but to ease the lecture of the table.

Standard major axis regressions:

Slopes and elevations of the relationships between the variables using the standardized major axis (SMA) line-fitting method with Huber's M estimation [43], are shown in table S5.

Table S5: Standar major axis (SMA) regression elevations and slopes.

	Body mass M (kg)		95 % CI	
l (mm)	slope	0.31	0.29	0.33
	elev.	1.70	1.67	1.73
	r^2: 0.94		P < .001	
L (mm)	slope	0.36	0.35	0.37
	elev.	0.90	0.88	0.93
	r^2: 0.97		P < .001	
D (mm)	slope	0.38	0.37	0.40
	elev.	0.60	0.58	0.63
	r^2: 0.97		P < .001	
$D_{max}/2$ (mm)	slope	0.38	0.37	0.39
	elev.	0.43	0.41	0.46
	r^2: 0.97		P < .001	
$D_{min}/2$ (mm)	slope	0.41	0.39	0.43
	elev.	0.09	0.05	0.12
	r^2: 0.94		P < .001	
ΔR (mm)	slope	0.36	0.34	0.38
	elev.	0.14	0.10	0.18
	r^2: 0.92		P < .001	
$\frac{\Delta R}{L}$	slope	-0.10	-0.12	-0.08
	elev.	-0.62	-0.66	-0.59
	r^2: 0.01		P = .425	
A (m^2)	slope	0.74	0.72	0.76
	elev.	-4.48	-4.52	-4.44
	r^2: 0.98		P < .001	
σ (Pa)	slope	0.11	0.09	0.14
	elev.	6.27	6.22	6.31
	r^2: 0.16		P < .001	
V $\frac{m}{s}$	slope	-0.08	-0.09	-0.06
	elev.	0.67	0.65	0.70
	r^2: 0.31		P < .001	

	l(mm)		95 % CI	
L (mm)	slope	1.17	1.12	1.22
	elev.	-1.09	-1.20	-0.98
	r^2: 0.95		P < .001	
D (mm)	slope	1.25	1.21	1.30
	elev.	-1.54	-1.64	-1.43
	r^2: 0.97		P < .001	
$D_{max}/2$ (mm)	slope	1.22	1.17	1.26
	elev.	-1.64	-1.74	-1.54
	r^2: 0.97		P < .001	
$D_{min}/2$ (mm)	slope	1.34	1.27	1.41
	elev.	-2.19	-2.35	-2.04
	r^2: 0.93		P < .001	
ΔR (mm)	slope	1.13	1.07	1.20
	elev.	-1.78	-1.92	-1.65
	r^2: 0.94		P < .001	
$\frac{\Delta R}{L}$	slope	-0.31	-0.39	-0.25
	elev.	-0.09	-0.23	0.06
	r^2: 1.5e-4		P = .893	
A (m^2)	slope	2.41	2.32	2.50
	elev.	-8.59	-8.79	-8.39
	r^2: 0.97		P < .001	
σ (Pa)	slope	0.38	0.31	0.47
	elev.	5.61	5.44	5.78
	r^2: 0.05		P = .013	
V $\frac{m}{s}$	slope	-0.25	-0.30	-0.21
	elev.	1.10	0.99	1.21
	r^2: 0.16		P < .001	

	L (mm)		95 % CI	
D (mm)	slope	1.07	1.04	1.10
	elev.	-0.36	-0.41	-0.32
	r^2: 0.98		P < .001	
$D_{max}/2$ (mm)	slope	1.05	1.02	1.09
	elev.	-0.52	-0.57	-0.47
	r^2: 0.98		P < .001	
$D_{min}/2$ (mm)	slope	1.14	1.09	1.19
	elev.	-0.94	-1.02	-0.86
	r^2: 0.94		P < .001	
ΔR (mm)	slope	0.98	0.93	1.04
	elev.	-0.75	-0.82	-0.67
	r^2: 0.94		P < .001	
$\frac{\Delta R}{L}$	slope	-0.27	-0.32	-0.22
	elev.	-0.39	-0.47	-0.31
	r^2: 0.01		P = .310	
A (m^2)	slope	2.06	2.03	2.10
	elev.	-6.36	-6.40	-6.31
	r^2: 0.99		P < .001	
σ (Pa)	slope	0.32	0.26	0.39
	elev.	5.98	5.88	6.07
	r^2: 0.06		P = .006	
V $\frac{m}{s}$	slope	-0.21	-0.25	-0.18
	elev.	0.86	0.81	0.92
	r^2: 0.26		P < .001	

	D (mm)		95 % CI	
$D_{max}/2$ (mm)	slope	0.98	0.97	0.99
	elev.	-0.16	-0.17	-0.14
	r^2: 0.99		P < .001	
$D_{min}/2$ (mm)	slope	1.07	1.04	1.10
	elev.	-0.55	-0.59	-0.52
	r^2: 0.98		P < .001	
ΔR (mm)	slope	0.92	0.87	0.97
	elev.	-0.41	-0.47	-0.35
	r^2: 0.94		P < .001	
$\frac{\Delta R}{L}$	slope	-0.25	-0.30	-0.20
	elev.	-0.48	-0.54	-0.41
	r^2: 2.3e-3		P = .603	
A (m^2)	slope	1.93	1.90	1.96
	elev.	-5.65	-5.69	-5.62
	r^2: 0.99		P < .001	
σ (Pa)	slope	0.30	0.24	0.36
	elev.	6.09	6.01	6.17
	r^2: 0.06		P = .006	
V $\frac{m}{s}$	slope	-0.20	-0.23	-0.16
	elev.	0.79	0.74	0.84
	r^2: 0.17		P < .001	

	$D_{max}/2$ (mm)		95 % CI	
$D_{min}/2$ (mm)	slope	1.09	1.06	1.13
	elev.	-0.39	-0.43	-0.35
	r^2: 0.97		P < .001	
ΔR (mm)	slope	0.94	0.90	0.98
	elev.	-0.26	-0.31	-0.22
	r^2: 0.96		P < .001	
$\frac{\Delta R}{L}$	slope	-0.25	-0.31	-0.21
	elev.	-0.52	-0.58	-0.46
	r^2: 3.0e-05		P = .953	
A (m^2)	slope	1.97	1.93	2.01
	elev.	-5.34	-5.38	-5.30
	r^2: 0.99		P < .001	
σ (Pa)	slope	0.30	0.24	0.37
	elev.	6.13	6.06	6.21
	r^2: 0.06		P = .007	
V $\frac{m}{s}$	slope	-0.20	-0.24	-0.17
	elev.	0.76	0.72	0.80
	r^2: 0.17		P < .001	

	$D_{min}/2$ (mm)		95 % CI	
ΔR (mm)	slope	0.86	0.80	0.93
	elev.	0.07	0.01	0.12
	r^2: 0.87		P < .001	
$\frac{\Delta R}{L}$	slope	-0.23	-0.28	-0.19
	elev.	-0.61	-0.65	-0.57
	r^2: 0.02		P = .187	
A (m^2)	slope	1.81	1.75	1.87
	elev.	-4.66	-4.71	-4.61
	r^2: 0.97		P < .001	
σ (Pa)	slope	0.27	0.22	0.33
	elev.	6.25	6.20	6.30
	r^2: 0.06		P = .008	
V $\frac{m}{s}$	slope	-0.18	-0.22	-0.15
	elev.	0.69	0.66	0.72
	r^2: 0.14		P < .001	

	ΔR (mm)		95 % CI	
$\frac{\Delta R}{L}$	slope	0.27	0.22	0.33
	elev.	-0.95	-1.00	-0.91
	r^2: 0.02		P = .110	
A (m^2)	slope	2.10	2.00	2.20
	elev.	-4.79	-4.87	-4.71
	r^2: 0.95		P < .001	
σ (Pa)	slope	0.33	0.27	0.41
	elev.	6.22	6.16	6.27
	r^2: 0.06		P = .009	
V $\frac{m}{s}$	slope	-0.21	-0.26	-0.18
	elev.	0.70	0.67	0.74
	r^2: 0.18		P < .001	

	$\Delta R/L$		95 % CI	
A (m^2)	slope	-7.79	-9.52	-6.38
	elev.	-9.38	-10.61	-8.15
	r^2: 0.005		P = .448	
σ (Pa)	slope	-1.25	-1.54	-1.02
	elev.	5.47	5.27	5.67
	r^2: 0.002		P = .620	
V $\frac{m}{s}$	slope	0.85	0.69	1.04
	elev.	1.21	1.08	1.35
	r^2: 0.03		P = .080	

	A(m^2)		95 % CI	
σ (Pa)	slope	0.15	0.12	0.19
	elev.	6.95	6.84	7.06
	r^2: 0.06		P = .006	
V $\frac{m}{s}$	slope	-0.10	-0.12	-0.09
	elev.	0.22	0.15	0.28
	r^2: 0.19		P < .001	

	σ (Pa)		95 % CI	
V $\frac{m}{s}$	slope	-0.66	-0.72	-0.61
	elev.	4.82	4.45	5.20
	r^2: 0.81		P < .001	

- l: the length of the humerus.
- L: the width of the distal articular surface of the humerus.
- D: average diameter of the distal articular surface of the humerus.
- $D_{max}/2$: maximum radius of the distal articular surface of the humerus.
- $D_{min}/2$: minimum radius of the distal articular surface of the humerus.
- ΔR: arithmetic difference between $D_{max}/2$ and $D_{min}/2$.
- A: projected area of the distal articular surface of the humerus calculated as $D \times L$.
- σ: average contact stress at the distal articular surface of the humerus.
- V: sliding speed at the distal articular surface of the humerus.
- Columns colors are not related to the values of the data but to ease the lecture of the table.

Landmarks positioning

The list of the common anatomical landmarks and semilandmarks used to determine the dimensions of the distal humerus are shown in table S6 and table S7, respectively.

The semilandmarks start and end at anatomical landmarks (fig. S5a), and were sub-sampled with at least 20 points (fig. S5b).

Table S6: Definition of the anatomical landmarks

**LM**	**Definition**
LM-1	Most disto-medial point of the trochlea
LM-2	Most medio-proximal point of the caudal side of the trochlea
LM-3	Most latero-proximal point of the caudal side of the trochlea
LM-4	Most distal point of contact between the trochlea and the capitulum
LM-5	Most latero-proximal point of the cranial side of the capitulum
LM-6	Maximum concavity of the cranial margin of the trochlea point
LM-7	Most medio-proximal point of the cranial side of the trochlea
LM-8	Most proximal point of the greater trochanter

Table S7: Definition of the anatomical semilandmarks

**SLM**	**initial landmark**	**final landmark**
C-1	LM-1	LM-2
C-2	LM-2	LM-3
C-3	LM-3	LM-4
C-4	LM-4	LM-5
C-5	LM-5	LM-6
C-6	LM-6	LM-7
C-7	LM-7	LM-1

The length of the bones, l, was determined by measuring the distance between landmarks LM-1 and LM-8 as illustrated in fig. S5a. We extracted the articular surface of the distal humerus by selecting the bone surface within the semilandmarks shown in table S7 and transformed it into a point cloud (fig. S5b and fig. S6). We then fitted a cylinder to the point cloud using a Python subroutine (see [29])[3].

[3]available at https://doi.org/10.5281/zenodo.7993776

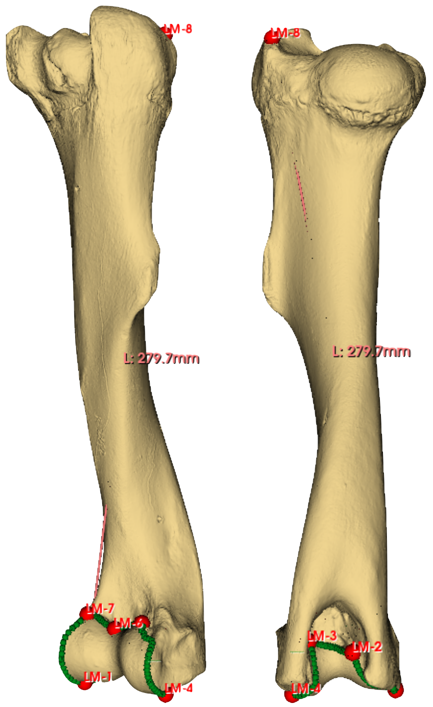

(a) Landmarks (LM) and semilandmarks curves (C) placed on the bone.

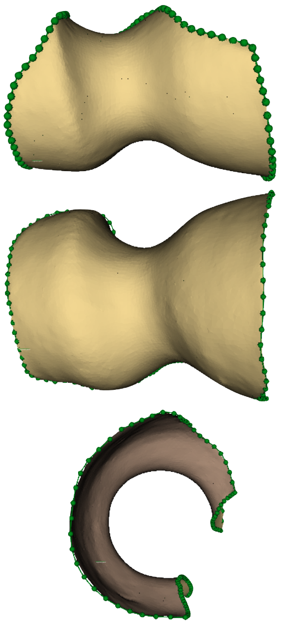

(b) Extracted articular surface.

Figure S5: The landmarks (LM), shown in red, and semilandmarks (C), in green, placed on the humerus of a *Lama glama* (Object: imnh:r:2392, Media ID: 000120933 from MorphoSource.org). The curves of the semilandmarks demarcate the articular surface.

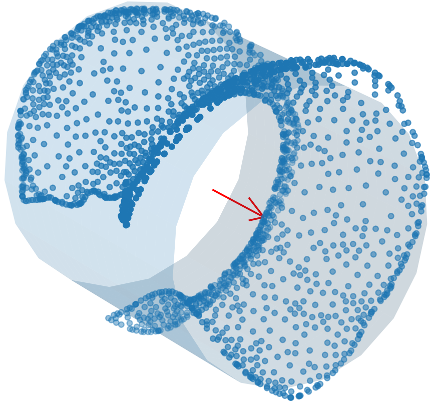

Figure S6: Cylinder fitted to the cloud of points generated from the articular surface with the central axis of the cylinder is shown as a red arrow.

Silhouette credits

Silhouettes are from PhyloPic; from left to right in the figure:

- *Didelphis virginiana*, Public Domain Dedication
- *Vombatus ursinus*, Public Domain Dedication
- *Acrobates pygmaeus* by Sarah Werning, CC BY 3.0
- *Isoodon macrourus*, Public Domain Dedication
- *Myrmecobius fasciatus*, by Sarah Werning (modified), CC BY 3.0
- *Sarcophilus harrisii*, Public Domain Dedication
- *Elephas maximus*, Public Domain Dedication
- *Orycteropus afer*, Public Domain Mark 1.0
- *Ptilocercus lowii*, Public Domain Dedication
- *Castor canadensis*, Public Domain Dedication
- *Hydrochoerus hydrochaeris*, Public Domain Dedication
- *Genetta genetta*, Public Domain Mark 1.0
- *Panthera leo*, Public Domain Dedication
- *Canis lupus*, Public Domain Dedication
- *Acinonyx jubatus*, by Gabriela Palomo-Munoz, CC BY-NC 3.0
- *Ursus arctos*, Public Domain Dedication
- *Equus caballus*, Public Domain Dedication
- *Diceros bicornis*, Public Domain Dedication
- *Giraffa camelopardalis*, Public Domain Dedication
- *Antilocapra americana*, Public Domain Dedication
- *Syncerus caffer*, Public Domain Dedication
- *Bos bison*, Public Domain Dedication
- *Kobus ellipsiprymnus*, by Jan A. Venter, Herbert H. T. Prins, David A. Balfour & Rob Slotow (vectorized by T. Michael Keesey), CC BY 3.0
- *Antidorcas marsupialis*, by Robert Hering, CC BY 3.0
- *Cephalophus silvicultor*, by Kai Caspar, CC BY 4.0
- *Hippotragus equinus*, Public Domain Dedication
- *Connochaetes taurinus*, by Lukasiniho CC BY-NC-SA 3.0
- *Ovibos moschatus*, Public Domain Dedication
- *Rupicapra rupicapra*, Public Domain Dedication
- *Ovis aries*, by Gabriela Palomo-Munoz, CC BY-NC 3.0

Clasical theories of allometry

Our allometries for contact stress and sliding speed are also supported by the three classical theories of allometry. These theories suggest that animals scale to preserve either geometric similarity (lengths, $l \propto M^{1/3}$, and widths, r, $\propto M^{1/3}$), elastic similarity ($l \propto M^{1/4}$, and $r \propto M^{3/8}$), or constant stress similarity ($l \propto M^{1/5}$, and $r \propto M^{2/5}$) [30]. The predicted allometric exponents of motion quantities for a generic hinge-like joint related to each theory are shown in Table table S8. All the quantities were determined similarly as in the result section, except for the excursion time, t. As applying allometry within the three similarity rules framework to stride frequency during galloping is not straightforward [30], t was defined with the excursion angle, θ (proportional to muscle length and inversely proportional to joint radius). Our analysis revealed that regardless of the similarity rule, the consistency of the contact stress and sliding speed remains.

Table S8: Allometric exponents of motion quantities for a generic hinge-like joint.

		Allometric exponent		
Parameter	Expression[a]	Geometric	Elastic	Constant stress
Moments of inertia	$I \propto mr^2$	5/3	7/4	9/5
Maximum muscular Force	$F \propto r^2$	2/3	3/4	4/5
Muscle moment arm	$k \propto r$	1/3	3/8	2/5
Angular acceleration	$\alpha = Fk/I$	−2/3	−5/8	−3/5
Excursion angle	$\theta \propto l/r$	0.0	−1/8	−1/5
Excursion time	$t \propto (\theta/\alpha)^{1/2}$	1/3	1/4	1/5
Angular speed	$\omega = \alpha t$	−1/3	−3/8	−2/5
Spatially averaged contact stress	$P = F/r^2$	0.0	0.0	0.0
Potential top sliding speed	$v_{sup} = \omega r$	0.0	0.0	0.0

[a] l, are lengths distances; r, are radial distances.

Lubrication regime

In this section, we analyze the lubricant film thickness of the elbow joint. Then, we use this lubricant film thickness and the rugosity of the cartilage to determine the film parameter that gives the lubrication regime of the elbow. Finally, we develop an analysis of how the sliding speed affects the lubrication regime.

Film thickness

We start the analysis by assuming the elbow as two cylindrical surfaces in conformal contact. The internal cylinder has a diameter of D^*, whereas the external cylinder of D_u (fig. S7).

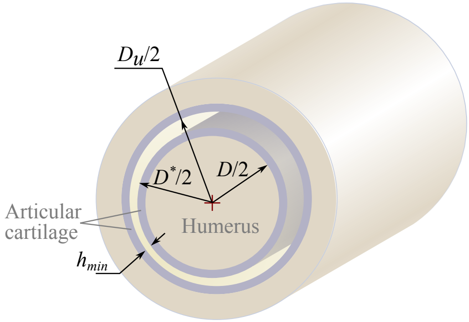

Figure S7: simplfication of the elbow as two cylinders in conformal contact

As the thickness of the cartilage is assumed to be constant across the articular surface, and $D_u \propto D$, we have:

$$D^* = D(1 + c_i) \tag{3}$$

$$D_u = D(1 + c_i + c_{ii}) \tag{4}$$

where c_i and c_{ii} are proportion parameters that define the articular cartilage thickness and the difference in diameter of the two surfaces, respectively.

The minimum lubricant film thickness (h_{min}) is defined as:

$$h_{min} = H_{min} R_x \tag{5}$$

where H_{min} is the dimensionless film thickness and R_x is the effective radius for two conformal cylinders in contact:

$$R_x = \frac{1}{2/D^* - 2/D_u} \tag{6}$$

For isoviscous-elastic lubrication, H_{min} is defined as [15]:

$$H_{min} = 7.43 U^{0.65} W^{-0.21} (1 - 0.85 e^{-0.31k}) \tag{7}$$

where U is the dimensionless speed parameter, W is the dimensionless load parameter, and k is the ellipticity parameter (ratio between the diameters of the contact zone ellipse). For conformal cylindrical surfaces in contact $k > 36$, so the last term in eq. (7) is negligible [15].

The dimensionless parameters U and W are defined as follows:

$$U = \frac{\eta V^*}{E' R_x} \tag{8}$$

$$W = \frac{F}{E' R_x^2} \tag{9}$$

Here, η is the viscosity, F the normal load, V^* the sliding speed at the diameter D^* (eq. (10)), and E' the effective Young's modulus calculated using the cartilage Young's modulus (E) and Poisson's ratio (ν) (eq. (11)).

$$V^* = \frac{V D^*}{D} \tag{10}$$

$$E' = \frac{E}{1 - \nu^2} \tag{11}$$

We used the allometric expression for the triceps force, $F_m = 78.20M^{0.78}$ (F_m is in N and M is in kg) [3, 34], to determine the normal force, $F = F_m$. By replacing all the terms in eq. (5) and eq. (7) and using the appropriate allometric expressions for V, D, F_m (table S9), we have:

$$h_{min} = \left[\frac{4.36D_a^{0.77}\eta^{0.65}V_a^{0.65}}{E'^{0.44}F_a^{0.21}}\right]\left[\frac{(c_i+1)^{1.42}(c_{ii}+c_i+1)^{0.77}}{c_{ii}^{0.77}}\right]M^{0.06} \tag{12}$$

Here, the terms with subindex a are the allometric coefficients of the parameters. As the allometric exponent for h_{min} is < 0.1, the lubricant film thickness is considered consistent across the analyzed taxa.

The first term in eq. (12) is determined with the values shown in table S9. Hence, we can analyze h_{min} regarding the chosen values for c_i and c_{ii}. In fig. S8 is shown the h_{min} as a function of c_i and c_{ii} within a typical physiological range for the elbow ($0.00 < c_i \leq 0.1$ and $0.01 < c_{ii} \leq 0.1$). By analyzing eq. (12), h_{min} appears unaffected for $c_{ii} > 10$, however, for $c_{ii} < 2$, h_{min} increments inversely proportional to c_{ii}. Conversely, h_{min} increments with c_i.

Table S9: Values for the parameters used for h_{min}

Parameter, Z		Coefficient, Z_a	Allometric exponent, Z_b	Ref
Average diameter, mm	D	$10^{0.6}$	0.35	table S4
Young's modulus, MPa	E	8.1	0.0	[33]
Poisson's coef.	ν	0.4	0.0	[21]
Viscosity, Pa·s	η	0.01	0.0	[25, 26]
Sliding speed, m/s	V	$10^{0.61}$	-0.07	table S4
Normal force, N	F_m	78.20	0.78	[3, 34]

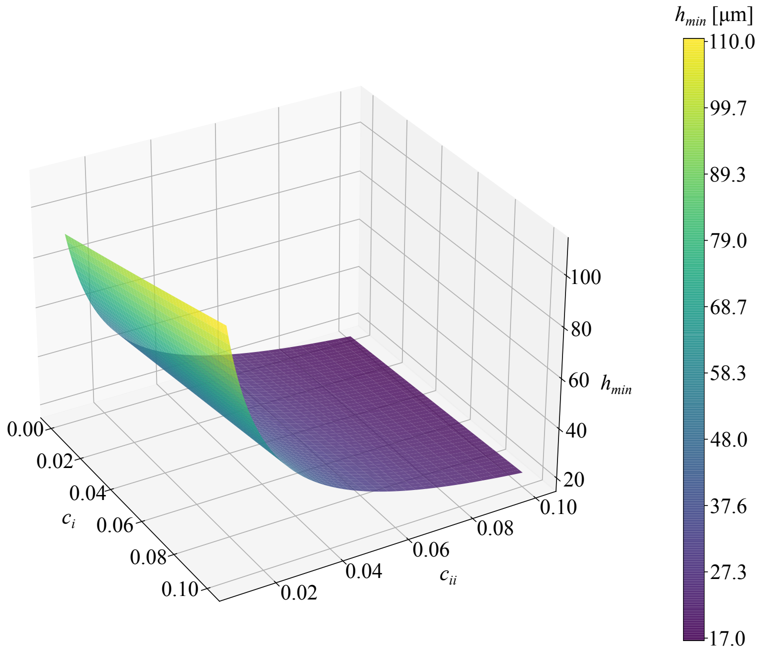

Figure S8: Variation of the minimum film thickness, h_{min}, as a function of the parameters c_i and c_{ii} within physiological ranges.

Film parameter

The lubrication regime of two bodies in contact is classified based on the film parameter Λ, which compares the minimum thickness of the lubricant to the combined roughness of the contacting surfaces, R_a and $R_{a,u}$ (eq. (13)) [15]. Since both surfaces are covered with cartilage, the roughnesses are equal ($R_a = R_{a,u}$). The classical classification of the lubrication regime is shown in table S10.

$$\Lambda = \frac{h_{min}}{\sqrt{R_a^2 + R_{a,u}^2}} \tag{13}$$

We evaluate Λ over a broad range of R_a, c_i, and c_{ii} values (fig. S9). The results show that for more conformal surfaces with $c_{ii} < 1$, the lubrication regime remains within fluid film lubrication (either elastohydrodynamic or hydrodynamic), even when the surface roughness increases substantially, such as in the case of osteoarthritis (fig. S9).

Table S10: Classical classification of the lubrication regime in engineering.

Boundary lubrication	$\Lambda < 1$		There is contact between the asperities of the surfaces.
Mixed lubrication	$1 \leq \Lambda < 3$		Some contact will take place between the asperities.
Fluid film	$3 \leq \Lambda < 5$	Elastohydrodynamic lubrication	The relative motion of the surfaces causes a positive pressure (lifting), which keeps a film of lubricant between the surfaces. There are significant deformations of the surfaces.
	$\Lambda \geq 5$	Hydrodynamic lubrication	The surfaces in contact are also separated by a thin film of lubricant but the deformations of the surfaces in contact are smaller compared to the elastohydrodynamic regime.

The roughness of cartilage falls within the range of 0.086 - 6.5 µm [11, 13, 18, 20, 24–26, 37–39, 42]. To assess the range of values of Λ under physiological conditions, we evaluated it for various combinations of R_a, c_i, and c_{ii} (fig. S10-a). The results show that the fluid film regime is always maintained, except for instances of large roughness and less conformal geometries.

We estimated a lubrication regime for the elbow by assigning values of c_i and c_{ii} based on reported values for the ankle joint by Medley et al. [31], which gives $c_i = 0.06$ and $c_{ii} = 0.07$. For the surface roughness, we used the recent value proposed by Liao et al. [25, 26] of $R_a = 1.42$ µm. Based on these values, we obtained $h_{min} = 24.16$ µm and $\Lambda = 12.03 \gg 3$. Hence, the elbow operates in the fluid film lubrication regime.

Studies have shown that micro-elastohydrodynamic effects occur on soft surfaces, such as cartilage. This effect is characterized by the smoothing of surface asperities due to local pressures, increasing the film thickness relative to the 'effective' roughness of the surfaces [11]. As a result, fluid film lubrication can occur for $\Lambda \geq 1$ [16]. As per the micro-elastohydrodynamic effect (see table S11), fluid film lubrication can be achieved for near-physiological ranges (fig. S10-b).

Table S11: Classification of the lubrication regime in engineering considering the micro-elastohydrodynamic effect.

$\Lambda < 0.1$	Boundary lubrication
$0.1 \leq \Lambda < 1$	Mixed lubrication
$1 \leq \Lambda < 5$	Elastohydrodynamic lubrication
$\Lambda > 5$	Hydrodynamic lubrication

Sliding speed

To account for the uncertainties due to the assumptions made to analyze the sliding speed, we evaluated Λ for a wide range of V and R_a (fig. S11). The results show that the elbow joint still operates in the fluid film regime. Nevertheless, for high surface roughness and low sliding velocities, the lubrication regime can shift towards boundary lubrication. It is worth noting that for slow movements, the sliding speed may be influenced by the mass, and other properties of the cartilage have an effect on the lubrication, e.g. the squeeze-film action or surface properties.

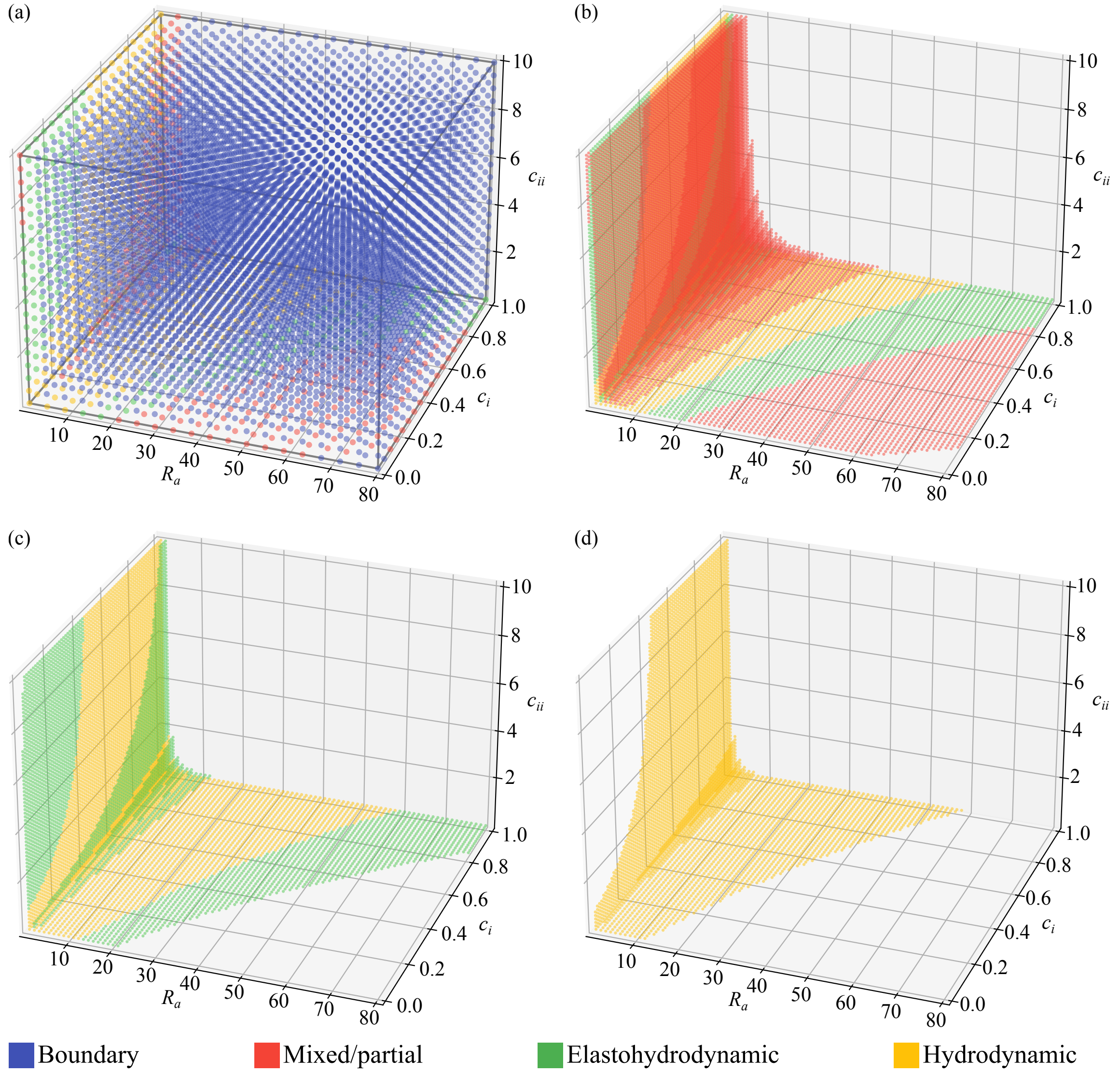

Figure S9: Lubrication regime as a function of the cartilage rugosity R_a, and the parameters c_i and c_{ii}. (a) all the regimes are shown; (b) without the boundary lubrication regime; (c) without the boundary and mixed lubrication; (d) only the hydrodynamic lubrication.

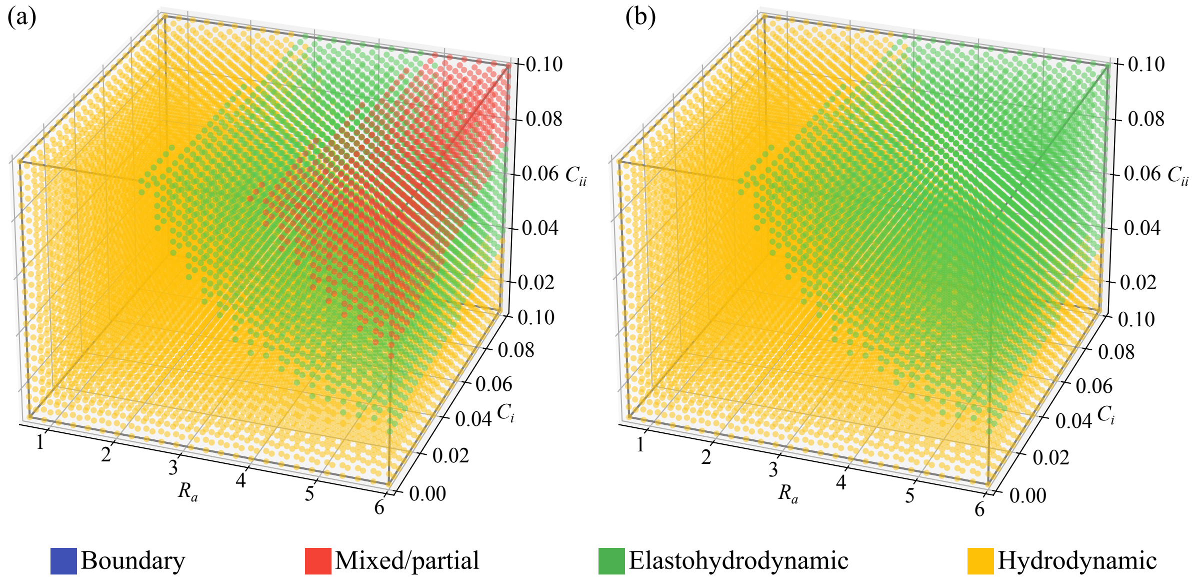

Figure S10: Cloud of points of the lubrication regime as a function of the cartilage rugosity R_a, and the parameters c_i and c_{ii}. (a) with the traditional classification of Λ; (b) with the classification of Λ that considers micro-elastohydrodynamic.

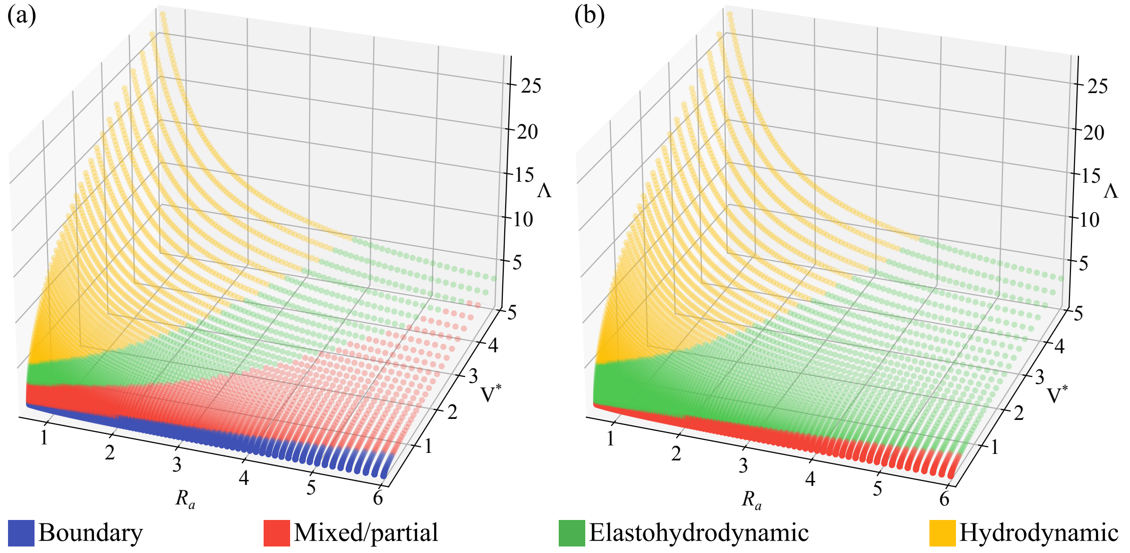

Figure S11: Λ and lubrication regime as a function of the speed V^* and the rugosity of the cartilage R_a: (a) with the traditional classification of Λ; (b) with the classification of Λ that considers micro-elastohydrodynamic.

References

[1] Plotdigitizer, 2022. URL https://plotdigitizer.com.

[2] Japanese marten, 2023. URL https://en.wikipedia.org/wiki/Japanese_marten.

[3] R. Mc N. Alexander, A. S. Jayes, G. M.O. Maloiy, and E. M. Wathuta. Allometry of the leg muscles of mammals. *Journal of Zoology*, 194(4):539–552, 1981. ISSN 14697998. doi: 10.1111/j.1469-7998.1981.tb04600.x.

[4] N Anna Toenjes. Felis catus, 2014. URL https://animaldiversity.org/accounts/Felis_catus/. (On-line), Animal Diversity Web.

[5] D. Armitage. Rattus norvegicus, 2004. URL https://animaldiversity.org/accounts/Rattus_norvegicus/. (On-line), Animal Diversity Web.

[6] Per Christiansen. Scaling of the limb long bones to body mass in terrestrial mammals. *Journal of Morphology*, 239(2):167–190, 1999. ISSN 03622525. doi: 10.1002/(SICI)1097-4687(199902)239:2⟨167::AID-JMOR5⟩3.0.CO;2-8.

[7] Per Christiansen. Locomotion in terrestrial mammals: the influence of body mass, limb length and bone proportions on speed. *Zoological Journal of the Linnean Society*, 136(4):685–714, dec 2002. ISSN 1096-3642. doi: 10.1046/j.1096-3642.2002.00041.x. URL https://academic.oup.com/zoolinnean/article-lookup/doi/10.1046/j.1096-3642.2002.00041.x.

[8] Samuel J Coatham, William I Sellers, and Thomas A Püschel. Convex hull estimation of mammalian body segment parameters. *Royal Society Open Science*, 8(6):210836, jun 2021. ISSN 20545703. doi: 10.1098/rsos.210836. URL https://royalsocietypublishing.org/doi/10.1098/rsos.210836.

[9] Samuel J Coatham, William I Sellers, and Thomas A Püschel. *Convex hull estimation of mammalian body segment parameters, Dryad, Dataset*, 2021.

[10] J. P. De MagalhÃes and J. Costa. A database of vertebrate longevity records and their relation to other life-history traits. *Journal of Evolutionary Biology*, 22(8):1770–1774, 2009. ISSN 1010061X. doi: 10.1111/j.1420-9101.2009.01783.x. URL https://genomics.senescence.info/species/.

[11] D Dowson and Zhong-min Jin. Micro-Elastohydrodynamic Lubrication of Synovial Joints. *Engineering in Medicine*, 15(2): 63–65, apr 1986. ISSN 0046-2039. doi: 10.1243/EMED_JOUR_1986_015_019_02. URL http://journals.sagepub.com/doi/10.1243/EMED_JOUR_1986_015_019_02.

[12] Arthur WM English. An Electromyographic Analysis of Forelimb Muscles during Overground Stepping in the Cat. *Journal of Experimental Biology*, 76(1):105–122, oct 1978. ISSN 1477-9145. doi: 10.1242/jeb.76.1.105. URL https://journals.biologists.com/jeb/article/76/1/105/22552/An-Electromyographic-Analysis-of-Forelimb-Muscles.

[13] M Gabriela Espinosa, Gaston A Otarola, Jerry C Hu, and Kyriacos A Athanasiou. Cartilage Assessment Requires a Surface Characterization Protocol: Roughness, Friction, and Function. *Tissue Engineering Part C: Methods*, 27(4):276–286, apr 2021. ISSN 1937-3384. doi: 10.1089/ten.tec.2020.0367. URL https://www.liebertpub.com/doi/10.1089/ten.tec.2020.0367.

[14] G E Goslow, H. J. Seeherman, C R Taylor, M. N. McCutchin, and N C Heglund. Electrical activity and relative length changes of dog limb muscles as a function of speed and gait. *Journal of Experimental Biology*, 94(1):15–42, oct 1981. ISSN 1477-9145. doi: 10.1242/jeb.94.1.15. URL https://journals.biologists.com/jeb/article/94/1/15/23105/Electrical-activity-and-relative-length-changes-of.

[15] B.J. Hamrock, B.J. Schmid, and B.O. Jacobson. *Fundamentals of Fluid Film Lubrication*. Mechanical engineering series. CRC Press, 2004. ISBN 9780203021187. URL https://books.google.fr/books?id=s_sTzyB2QmYC.

[16] Jonny Hansen, Marcus Björling, and Roland Larsson. A New Film Parameter for Rough Surface EHL Contacts with Anisotropic and Isotropic Structures. *Tribology Letters*, 69(2):37, jun 2021. ISSN 1023-8883. doi: 10.1007/s11249-021-01411-3. URL https://doi.org/10.1007/s11249-021-01411-3https://link.springer.com/10.1007/s11249-021-01411-3.

[17] V.G. Heptner and N.P. Naumov. *Sirenia and Carnivora (Sea cows; Wolves, and Bears)*, volume II of *Mammals of the Soviet Union*. Science Publishers, Inc., USA, 1998. ISBN 1-886106-81-9.

[18] Mikhail Ihnatouski, Jolanta Pauk, Dmitrij Karev, and Boris Karev. AFM-Based Method for Measurement of Normal and Osteoarthritic Human Articular Cartilage Surface Roughness. *Materials*, 13(10):2302, may 2020. ISSN 1996-1944. doi: 10.3390/ma13102302. URL www.mdpi.com/journal/materialshttps://www.mdpi.com/1996-1944/13/10/2302.

[19] José Iriarte-Díaz. Differential scaling of locomotor performance in small and large terrestrial mammals. *Journal of Experimental Biology*, 205(18):2897–2908, 2002. ISSN 00220949. doi: 10.1242/jeb.205.18.2897.

[20] Z M Jin and D Dowson. Elastohydrodynamic Lubrication in Biological Systems. *Proceedings of the Institution of Mechanical Engineers, Part J: Journal of Engineering Tribology*, 219(5):367–380, may 2005. ISSN 1350-6501. doi: 10.1243/135065005X33982. URL http://journals.sagepub.com/doi/10.1243/135065005X33982.

[21] Wassif Kabir, Claudia Di Bella, Peter F.M. Choong, and Cathal D. O'Connell. Assessment of native human articular cartilage: A biomechanical protocol. *CARTILAGE*, 13(2_suppl):427S–437S, 2021. doi: 10.1177/1947603520973240. URL https://doi.org/10.1177/1947603520973240. PMID: 33218275.

[22] N. Kaminski. Leopardus geoffroyi, 2017. URL https://animaldiversity.org/accounts/Leopardus_geoffroyi/. (On-line), Animal Diversity Web.

[23] K. Kokx. Hemicentetes semispinosus, 2009. URL https://animaldiversity.org/accounts/Hemicentetes_semispinosus/. (On-line), Animal Diversity Web.

[24] Jinjing Liao, David W Smith, Saeed Miramini, Namal Thibbotuwawa, Bruce S Gardiner, and Lihai Zhang. The investigation of fluid flow in cartilage contact gap. *Journal of the Mechanical Behavior of Biomedical Materials*, 95:153–164, jul 2019. ISSN 17516161. doi: 10.1016/j.jmbbm.2019.04.008. URL www.elsevier.com/locate/jmbbmhttps://linkinghub.elsevier.com/retrieve/pii/S1751616118317843.

[25] Jinjing Liao, Saeed Miramini, Xuanchi Liu, and Lihai Zhang. Computational study on synovial fluid flow behaviour in cartilage contact gap under osteoarthritic condition. *Computers in Biology and Medicine*, 123(June):103915, 2020. ISSN 0010-4825. doi: 10.1016/j.compbiomed.2020.103915. URL https://doi.org/10.1016/j.compbiomed.2020.103915.

[26] Jinjing Liao, David W Smith, Saeed Miramini, Bruce S Gardiner, and Lihai Zhang. Investigation of role of cartilage surface polymer brush border in lubrication of biological joints. *Friction*, 10(1):110–127, 2022. ISSN 2223-7690. doi: 10.1007/s40544-020-0468-y. URL https://doi.org/10.1007/s40544-020-0468-y.

[27] Joerg-Henner Lotze and Sydney Anderson. Procyon lotor. *Mammalian Species*, pages 1–8, 06 1979. ISSN 0076-3519. doi: 10.2307/3503959. URL https://doi.org/10.2307/3503959.

[28] B. Lundrigan and Baker S. Paguma larvata, 2003. URL https://animaldiversity.org/accounts/Paguma_larvata/. (On-line), Animal Diversity Web.

[29] Kalenia Marquez-Florez, Santiago Arroyave-Tobon, Loic Tadrist, and Jean-Marc Linares. Allometry of the distal humerus lubrication parameters, zenodo, June 2023. URL https://doi.org/10.5281/zenodo.7993776.

[30] T.A. McMahon. *Muscles, Reflexes, and Locomotion*. Number v. 10 in Muscles, Reflexes, and Locomotion. Princeton University Press, 1984. ISBN 9780691023762. URL https://books.google.fr/books?id=hotzCgAAQBAJ.

[31] J B Medley, D Dowson, and V Wright. Surface Geometry of the Human Ankle Joint. *Engineering in Medicine*, 12(1):35–41, jan 1983. ISSN 0046-2039. doi: 10.1243/EMED_JOUR_1983_012_008_02. URL http://journals.sagepub.com/doi/10.1243/EMED_JOUR_1983_012_008_02.

[32] Alvaro Mones and Juhani Ojasti. Hydrochoerus hydrochaeris. *Mammalian Species*, pages 1–7, 06 1986. ISSN 0076-3519. doi: 10.2307/3503784. URL https://doi.org/10.2307/3503784.

[33] Miika T Nieminen, Juha Töyräs, Mikko S Laasanen, Johanna Silvennoinen, Heikki J Helminen, and Jukka S Jurvelin. Prediction of biomechanical properties of articular cartilage with quantitative magnetic resonance imaging. *Journal of Biomechanics*, 37(3):321–328, 2004. ISSN 00219290. doi: 10.1016/S0021-9290(03)00291-4.

[34] C. M. Pollock and R. E. Shadwick. Allometry of muscle, tendon, and elastic energy storage capacity in mammals. *American Journal of Physiology - Regulatory Integrative and Comparative Physiology*, 266(3 35-3), 1994. ISSN 00029513. doi: 10.1152/ajpregu.1994.266.3.r1022.

[35] R Core Team. *R: A Language and Environment for Statistical Computing*. R Foundation for Statistical Computing, Vienna, Austria, 2022. URL https://www.R-project.org/.

[36] J. Riney. Meles anakuma, 2011. URL https://animaldiversity.org/accounts/Meles_anakuma/. (On-line), Animal Diversity Web.

[37] R.S. Sayles, T.R. Thomas, J. Anderson, I. Haslock, and A. Unsworth. Measurement of the surface microgeometry of articular cartilage. *Journal of Biomechanics*, 12(4):257–267, jan 1979. ISSN 00219290. doi: 10.1016/0021-9290(79)90068-X. URL https://linkinghub.elsevier.com/retrieve/pii/002192907990068X.

[38] V.K. Shekhawat, M.P. Laurent, C. Muehleman, and M.A. Wimmer. Surface topography of viable articular cartilage measured with scanning white light interferometry. *Osteoarthritis and Cartilage*, 17(9):1197–1203, sep 2009. ISSN 10634584. doi: 10.1016/j.joca.2009.03.013. URL https://linkinghub.elsevier.com/retrieve/pii/S106345840900082X.

[39] Parvathy Thampi, Suzanne M Tabbaa, Brian Johnstone, Marcus A Wimmer, Michel P Laurent, C. Wayne McIlwraith, and David D Frisbie. Surface topography as a tool to detect early changes in a posttraumatic equine model of osteoarthritis. *Journal of Orthopaedic Research*, 40(6):1349–1357, jun 2022. ISSN 0736-0266. doi: 10.1002/jor.25175. URL https://onlinelibrary.wiley.com/doi/10.1002/jor.25175.

[40] Mikihiko Tokuriki. Electromyographic and Joint-Mechanical Studies in Quadrupedal Locomotion : III. Gallop. *The Japanese Journal of Veterinary Science*, 36(2):121–132, 1974. ISSN 0021-5295. doi: 10.1292/jvms1939.36.121. URL http://www.jstage.jst.go.jp/article/jvms1939/36/2/36_2_121/_article/-char/ja/.

[41] Nathan S. Upham, Jacob A Esselstyn, and Walter Jetz. *VertLife*, 2022. URL http://vertlife.org/phylosubsets/.

[42] P. S. Walker, J Sikorski, D Dowson, M D Longfield, V Wright, and T Buckley. Behaviour of synovial fluid on surfaces of articular cartilage. A scanning electron microscope study. *Annals of the rheumatic diseases*, 28(1):1–14, 1969. ISSN 00034967. doi: 10.1136/ard.28.1.1. URL http://ard.bmj.com/.

[43] David I Warton, Ian J Wright, Daniel S Falster, and Mark Westoby. Bivariate line-fitting methods for allometry. *Biological Reviews*, 81(02):259, may 2006. ISSN 1464-7931. doi: 10.1017/S1464793106007007. URL http://doi.wiley.com/10.1017/S1464793106007007.

[44] David I Warton, Remko A Duursma, Daniel S Falster, and Sara Taskinen. smatr 3- an R package for estimation and inference about allometric lines. *Methods in Ecology and Evolution*, 3(2):257–259, apr 2012. ISSN 2041210X. doi: 10.1111/j.2041-210X.2011.00153.x. URL http://www.respond2articles.com/MEE/https://onlinelibrary.wiley.com/doi/10.1111/j.2041-210X.2011.00153.x.

[45] K. Wickline. Sus scrofa, 2004. URL https://animaldiversity.org/accounts/Sus_scrofa/. (On-line), Animal Diversity Web.
